# Enhanced Virus Detection and Metagenomic Sequencing in Patients with Meningitis and Encephalitis

**DOI:** 10.1128/mBio.01143-21

**Published:** 2021-08-31

**Authors:** Anne Piantadosi, Shibani S. Mukerji, Simon Ye, Michael J. Leone, Lisa M. Freimark, Daniel Park, Gordon Adams, Jacob Lemieux, Sanjat Kanjilal, Isaac H. Solomon, Asim A. Ahmed, Robert Goldstein, Vijay Ganesh, Bridget Ostrem, Kaelyn C. Cummins, Jesse M. Thon, Cormac M. Kinsella, Eric Rosenberg, Matthew P. Frosch, Marcia B. Goldberg, Tracey A. Cho, Pardis Sabeti

**Affiliations:** a Broad Institute of MIT and Harvard, Cambridge, Massachusetts, USA; b Division of Infectious Diseases, Massachusetts General Hospital, Boston, Massachusetts, USA; c Emory University School of Medicine, Atlanta, Georgia, USA; d Department of Neurology, Massachusetts General Hospital, Boston, Massachusetts, USA; e Harvard Medical School, Boston, Massachusetts, USA; f Harvard-MIT Program of Health Sciences and Technology, Cambridge, Massachusetts, USA; g Department of Pathology, Brigham and Women’s Hospital, Boston, Massachusetts, USA; h Department of Pediatrics, Harvard Medical School, Children’s Hospital, Boston, Massachusetts, USA; i Department of Neurology, Brigham and Women’s Hospital, Boston, Massachusetts, USA; j Division of Infectious Diseases, Brigham and Women’s Hospital, Boston, Massachusetts, USA; k Department of Pathology, Massachusetts General Hospital, Boston, Massachusetts, USA; l University of Iowa, Department of Neurology, Iowa City, Iowa, USA; m Department of Organismic and Evolutionary Biology, Harvard University, Cambridge, Massachusetts, USA; n Department of Immunology and Infectious Disease, Harvard T. H. Chan School of Public Health, Boston, Massachusetts, USA; o Howard Hughes Medical Institute, Chevy Chase, Maryland, USA; Virginia Polytechnic Institute and State University

**Keywords:** encephalitis, metagenomic sequencing, next-generation sequencing (NGS), meningitis, virus, hybrid capture, methylated DNA depletion

## Abstract

Meningitis and encephalitis are leading causes of central nervous system (CNS) disease and often result in severe neurological compromise or death. Traditional diagnostic workflows largely rely on pathogen-specific tests, sometimes over days to weeks, whereas metagenomic next-generation sequencing (mNGS) profiles all nucleic acid in a sample. In this single-center, prospective study, 68 hospitalized patients with known (*n* = 44) or suspected (*n* = 24) CNS infections underwent mNGS from RNA and DNA to identify potential pathogens and also targeted sequencing of viruses using hybrid capture. Using a computational metagenomic classification pipeline based on KrakenUniq and BLAST, we detected pathogen nucleic acid in cerebrospinal fluid (CSF) from 22 subjects, 3 of whom had no clinical diagnosis by routine workup. Among subjects diagnosed with infection by serology and/or peripheral samples, we demonstrated the utility of mNGS to detect pathogen nucleic acid in CSF, importantly for the Ixodes scapularis tick-borne pathogens Powassan virus, Borrelia burgdorferi, and Anaplasma phagocytophilum. We also evaluated two methods to enhance the detection of viral nucleic acid, hybrid capture and methylated DNA depletion. Hybrid capture nearly universally increased viral read recovery. Although results for methylated DNA depletion were mixed, it allowed the detection of varicella-zoster virus DNA in two samples that were negative by standard mNGS. Overall, mNGS is a promising approach that can test for multiple pathogens simultaneously, with efficacy similar to that of pathogen-specific tests, and can uncover geographically relevant infectious CNS disease, such as tick-borne infections in New England. With further laboratory and computational enhancements, mNGS may become a mainstay of workup for encephalitis and meningitis.

## INTRODUCTION

Meningitis and encephalitis are leading causes of central nervous system (CNS) disease, ranked as the 4th leading contributor to global neurological disability-adjusted life-years (1), often resulting in severe neurological compromise or death (2, 3). Traditional diagnostic workflows remain inefficient, requiring costly pathogen-specific diagnostics, serial cerebrospinal fluid (CSF) testing, and sometimes invasive surgical procedures. Despite these intensive diagnostic efforts, 40 to 60% of subjects with meningitis or encephalitis have no clear cause identified (2, 4–6).

Metagenomic next-generation sequencing (mNGS) offers a unique opportunity to circumvent some of these challenges. mNGS consists of unbiased sequencing of all nucleic acid in a sample followed by computational classification of reads to identify potential pathogens (7–9). This technique has successfully detected a range of pathogens, including bacteria (10–12), fungi (13), protozoa (14), and viruses (15–17), in subjects with CNS infection. mNGS is increasingly used as a clinical diagnostic test (18–20), and criteria for test performance have been described but not yet standardized (21–23).

In this study, we prospectively enrolled 68 patients with known or suspected CNS infection and performed mNGS from both RNA and DNA to identify pathogens. We focused laboratory and analysis methods on viral nucleic acid detection since viruses are the most common type of pathogen detected in CNS infection (4, 5, 24, 25). The goals of this study were to assess the utility of standard mNGS in identifying CNS pathogens and to examine enhanced laboratory techniques for improving analytical sensitivity, including hybrid capture (HC) of viral nucleic acid and methylated DNA depletion (MDD).

## RESULTS

### Clinical characteristics.

**FIG 1:**
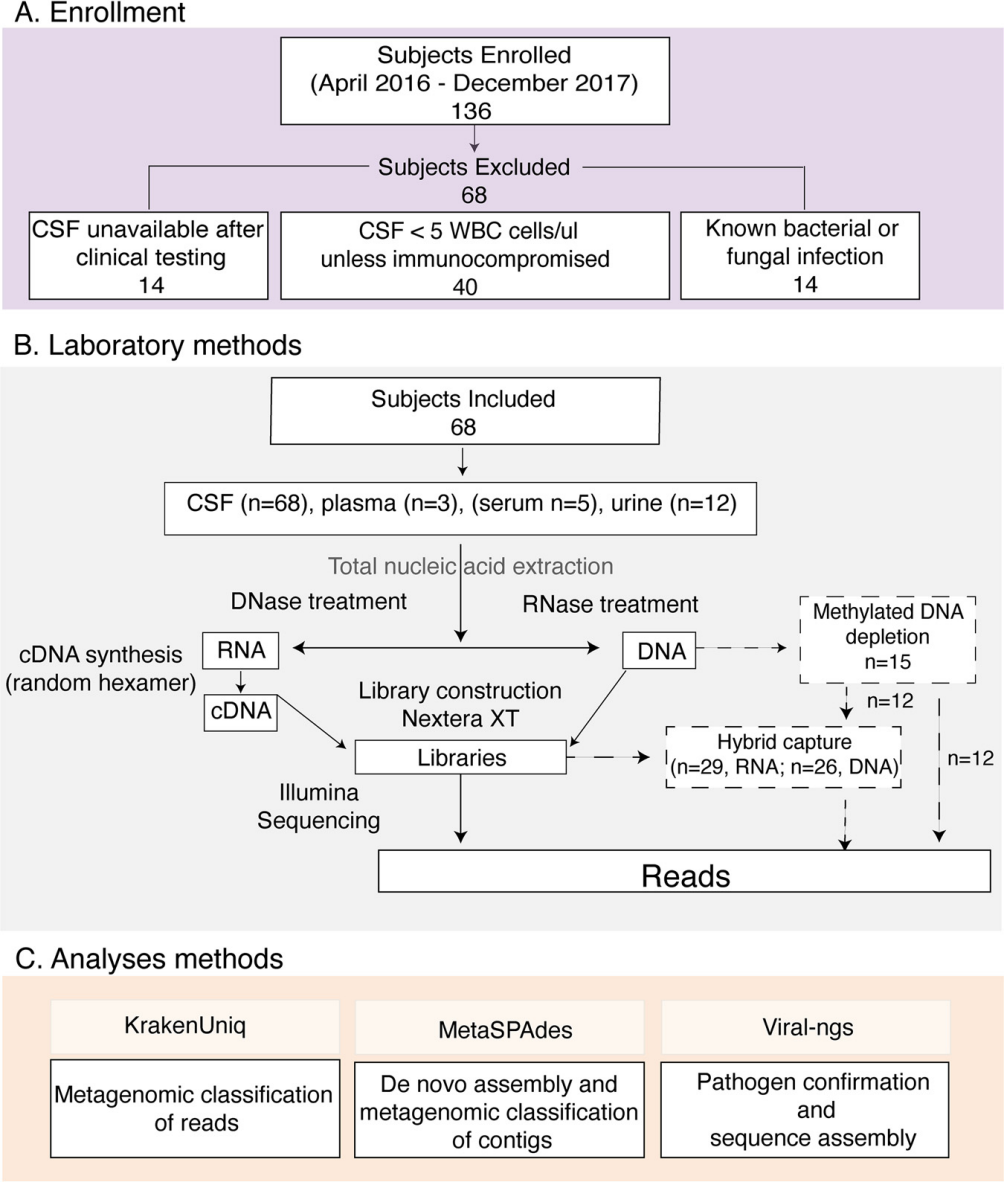
Overview of methods for subject selection and mNGS. Enrollment (A), laboratory methods (B), and analysis methods (C) are shown. Enhanced laboratory methods for methylated DNA depletion and hybrid capture (dashed lines) were included for a subset of the samples as shown. Abbreviations: CSF, cerebrospinal fluid; WBC, white blood cell.

Of the 68 adults enrolled, 63% (43/68) were male, subjects ranged in age from 24 to 86 years (median = 58 years [interquartile range {IQR}, 39, 72 years]) (Table 1), and 25 (37%) were immunocompromised (Fig. 1; see also Text S1 in the supplemental material). New England was the primary residence for all except one subject, who lived in Florida. Altered mental status was described in 56% (38/68), while a minority had photophobia (24% [16/68]) or neck stiffness (26% [18/68]). Twenty subjects out of 68 (29%) were admitted to the intensive care unit (ICU), and the in-hospital mortality rate was 6% (4/68).

**TABLE 1:** Clinical characteristics of enrolled subjects stratified by diagnostic group^*a*^

Characteristic	Value for group				
	Overall (*n* = 68)	Infection, PCR^+^ (*n* = 12)	Infection, other (*n* = 25)	Alternative diagnosis (*n* = 7)	Unknown (*n* = 24)
Demographics					
Median age (yrs) (IQR)	58.5 (39, 72.3)	57.5 (39, 67.3)	61 (43, 72)	73 (37.5, 77)	57.5 (38, 71)
No. of male subjects (%)	43 (63)	5 (42)	19 (76)	6 (86)	13 (54)
No. of immunocompetent subjects (%)	43 (63)	7 (58)	17 (68)	5 (71)	14 (58)
Median length of stay (days) (min, max)	8 (2, 51)	4.5 (2, 51)	9 (3, 51)	12 (3, 30)	7 (5, 22)

No. of subjects of race (%)					
White	57 (84)	10 (83.3)	21 (84)	6 (85.7)	20 (83.3)
Black or African American	2 (3)	1 (8)	0 (0)	1 (14)	0 (0)

No. of subjects with time from symptom onset to LP (%)					
Acute (0–3 days)	17 (25)	2 (16.7)	7 (28)	1 (14.3)	7 (29.2)
Early subacute (4–7 days)	12 (17.6)	5 (41.7)	3 (12)	1 (14.3)	3 (12.5)
Late subacute (8–30 days)	30 (44.1)	3 (25)	14 (56)	2 (28.6)	11 (45.8)
Chronic (>30 days)	9 (13.2)	2 (16.7)	1 (4)	3 (42.9)	3 (12.5)

Symptoms and signs during hospitalization					
No. of subjects with altered mental status (%)	38 (56)	7 (58.3)	14 (56)	5 (71.4)	12 (50)
No. of subjects with photophobia (%)	16 (24)	3 (25)	4 (16)	2 (28.6)	7 (29.2)
No. of subjects with neck stiffness (%)	18 (27)	2 (16.7)	6 (24)	2 (28.6)	8 (33.3)
Median max temp (°C) (IQR)	38.1 (37.4, 39)	37.8 (37.4, 38.1)	38.1 (37.6, 38.9)	37.7 (37.6, 39)	38.4 (37.4, 39.2)
No. of subjects with fever (max ≥ 38°C) (%)	38 (56)	6 (50)	14 (56)	3 (42.9)	15 (62.5)

Laboratory data					
Median laboratory parameter value (IQR)					
Hematology					
White blood cell count (WBCs/μI)	8.6 (7.4, 10.2)	8.8 (8.1, 9.6)	7.84 (6.5, 9.3)	8.7 (8.2, 9.7)	9.6 (7.5, 10.9)
CSF					
White blood cell count (WBCs/μI)	80.5 (16.8, 131.5)	105.5 (35.5, 337)	47 (14, 105)	17 (10, 25.5)	98.5 (40.5, 133.5)
Total protein (mg/dl)	70.5 (50, 117)	51.5 (39.8, 111)	65 (55, 117)	69 (44.5, 92.5)	78.5 (55, 120)
Glucose (mg/dl)	62 (54, 73.5)	67.5 (55, 82)	62 (55, 69)	60 (55, 64)	60 (52, 73.5)
Median no. of infectious disease tests ordered (min, max)	19 (6, 62)	12 (6, 56)	25 (6, 62)	26 (10, 57)	22.5 (6, 48)

No. of subjects with admission service (%)					
Medicine floor/ICU	21 (31)	4 (33.3)	9 (36)	0 (0)	8 (33.3)
Neurology floor/ICU	37 (54)	6 (50)	13 (52)	7 (100)	11 (45.8)
Other	10 (15)	2 (16.7)	3 (12)	0 (0)	5 (20.8)
Admission to ICU during hospitalization	20 (29)	3 (25)	9 (36)	3 (42.9)	5 (20.8)

No. of subjects admitted during study period (%)					
1 December–28 February	11 (16)	2 (16.7)	4 (16)	1 (14.3)	4 (16.7)
1 March–31 May	14 (21)	3 (25)	5 (20)	2 (28.6)	4 (16.7)
1 June–31 August	19 (28)	3 (25)	4 (16)	3 (42.9)	9 (37.5)
1 September–30 November	24 (35)	4 (33.3)	12 (48)	1 (14.3)	7 (29.2)

No. of subjects with postdischarge outcome (%)					
Home	39 (57)	5 (41.7)	12 (48)	3 (42.9)	19 (79.2)
Rehabilitation^*b*^	25 (37)	5 (41.7)	12 (48)	3 (42.9)	5 (20.8)
Death	4 (6)	2 (16.7)	1 (4)	1 (14.3)	0 (0)

a Abbreviations: CSF, cerebrospinal fluid; IQR, interquartile range; PCR^+^, positive PCR; ICU, intensive care unit.

b Long-term acute care or skilled nursing facility.

10.1128/mBio.01143-21.1 TEXT S1 Supplemental methods and results. Additional details regarding methods for participant enrollment, definitions used in the study, validation of sequencing results, and metagenomic data curation are included. The supplementary results include case vignettes. Download Text S1, DOCX file, 0.05 MB. Copyright © 2021 Piantadosi et al. 2021 Piantadosi et al. https://creativecommons.org/licenses/by/4.0/ This content is distributed under the terms of the Creative Commons Attribution 4.0 International license.

**FIG 2:**
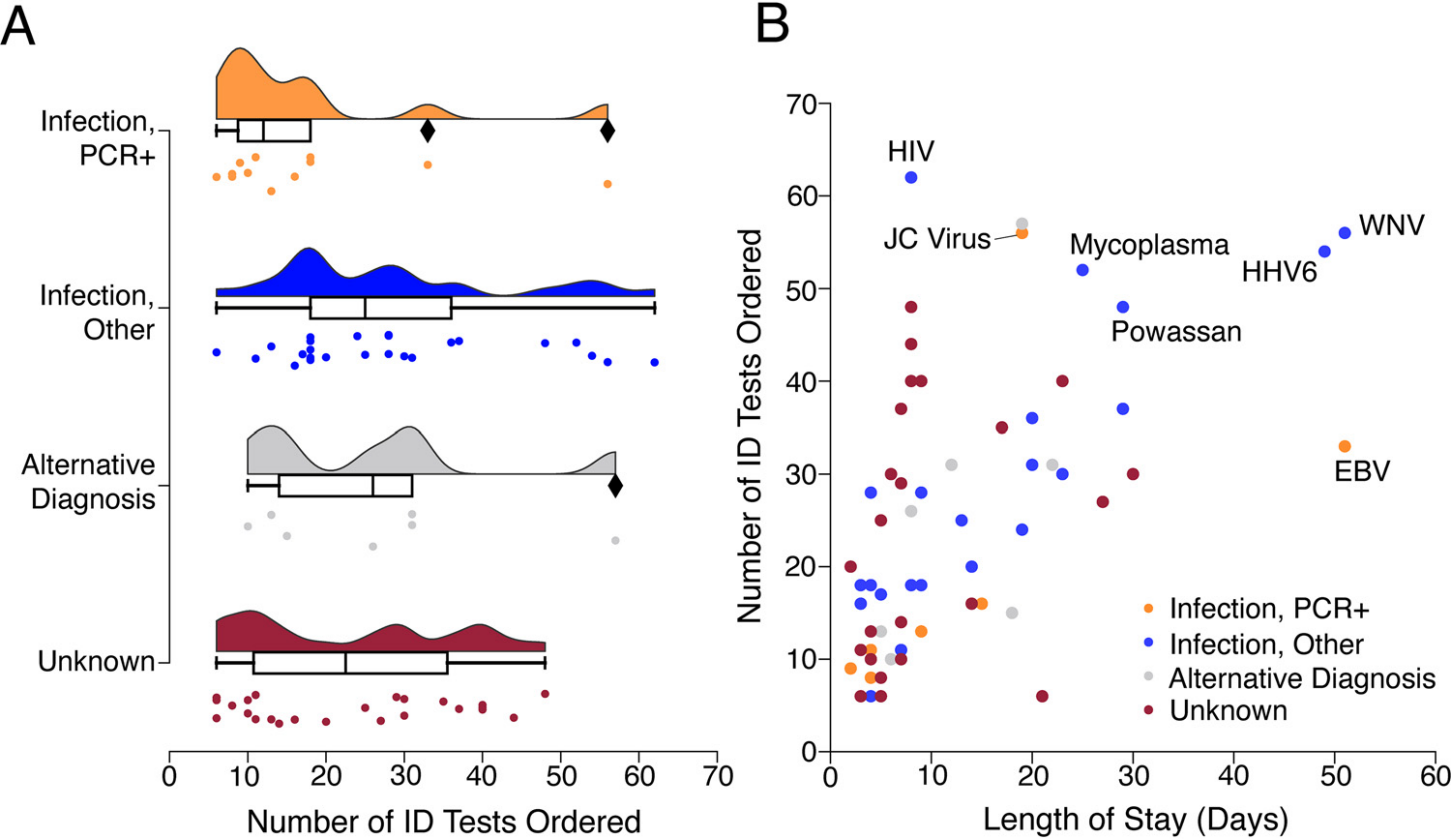
Number of infectious disease tests ordered and lengths of stay among subjects. (A) Distributions showing the number of infectious disease (ID)-related tests ordered per subject, stratified by clinical diagnosis category. ID tests were counted if ordered between hospital admission day 1 and hospital discharge. Box plots with horizontal bars represent medians and interquartile ranges for ID tests. Diamonds represent data points greater than 1.5× the IQR. (B) Scatterplot showing the number of ID tests versus length of stay per subject. Colors indicate clinical diagnosis categories. The LOS correlated with the number of total ID tests ordered (Spearman’s ρ = 0.65; *P* < 0.01). The final clinical diagnosis for viral pathogens is stated for cases whose number of ID tests or LOS was an outlier above the 3rd quartile.

Based on clinical testing, 44 of the 68 subjects received a conclusive diagnosis by discharge. Twelve subjects were diagnosed with viral infection by PCR from CSF (“infection, CSF PCR^+^” group), 25 were diagnosed with infection by serology or PCR from blood (“infection, other” group), and 7 had a noninfectious etiology (“alternative diagnosis” group). The remaining 24 subjects (35%) had no known diagnosis (“unknown” group) (Table 1). Subjects classified as “unknown” underwent exhaustive clinical testing; 50% of them (12/24) had ≥25 infectious disease (ID) tests (Fig. 2A; Fig. S2 and Table S2), and no diagnoses were made during long-term follow-up (Table S3). In contrast, the “infection, CSF PCR^+^” group had a much lower median number of clinical ID tests performed (12 [IQR, 6, 56] versus 22.5 [IQR, 11, 36] for the “unknown” group). The “infection, CSF PCR^+^” group also had the shortest length of stay (LOS) (4.5 days [IQR, 2, 51 days]), and across the total cohort, the LOS moderately correlated with the number of ID tests ordered (Spearman’s ρ = 0.65; *P* < 0.01) (Fig. 2B).

10.1128/mBio.01143-21.3 FIG S2 Correlations between the length of hospitalization and diagnostic testing ordered, stratified by clinical diagnosis. (A) Box plots showing the median length of stay (LOS) (horizontal line). Whiskers indicate the 1st and 3rd quartiles. Dots indicate an LOS greater than 1.5× the interquartile range. There were no significant differences in LOS between clinical diagnosis groups. (B and C) Scatterplots showing the number of CSF tests versus LOS (B) and the number of PCRs versus LOS (C). Colors indicate clinical diagnosis categories. The LOS moderately correlated with the number of total ID tests ordered (Spearman’s ρ = 0.65; *P* < 0.01) (Fig. 1B) and with the number of tests ordered from CSF only (Spearman’s ρ = 0.46; *P* < 0.01) (B). Download FIG S2, SVG file, 0.1 MB. Copyright © 2021 Piantadosi et al. 2021 Piantadosi et al. https://creativecommons.org/licenses/by/4.0/ This content is distributed under the terms of the Creative Commons Attribution 4.0 International license.

10.1128/mBio.01143-21.9 TABLE S2 Subject-level clinical details. Clinical details include cerebrospinal fluid white blood cells, duration of infection prior to lumbar puncture, and treatment provided for the 68 subjects in the study. The number of infectious disease tests includes tests performed during MGH hospitalization; infectious disease tests performed prior to an MGH admission were not included. Abbreviations: ALL, acute lymphocytic leukemia; ANCA, antineutrophil cytoplasmic antibodies; CLL, chronic lymphocytic leukemia; CNS, central nervous system; CVID, combined variable immunodeficiency; DLBCL, diffuse large B-cell lymphoma; GAD-65, glutamic acid decarboxylase 65-kDa isoform; HIV, human immunodeficiency virus; HSV, herpes simplex virus; HHV-6, human herpesvirus 6; ID, infectious disease; anti-NMDA-R, anti-*N*-methyl-d-aspartate receptor; PML, progressive multifocal leukoencephalopathy; WNV, West Nile virus; VZV, varicella-zoster virus. Download Table S2, XLSX file, 0.01 MB. Copyright © 2021 Piantadosi et al. 2021 Piantadosi et al. https://creativecommons.org/licenses/by/4.0/ This content is distributed under the terms of the Creative Commons Attribution 4.0 International license.

10.1128/mBio.01143-21.10 TABLE S3 Clinical follow-up for subjects listed as unknown at discharge. Chart review data for subjects classified as unknown at hospital discharge are shown. Download Table S3, XLSX file, 0.01 MB. Copyright © 2021 Piantadosi et al. 2021 Piantadosi et al. https://creativecommons.org/licenses/by/4.0/ This content is distributed under the terms of the Creative Commons Attribution 4.0 International license.

### Results from mNGS and enhanced methods.

**FIG 3:**
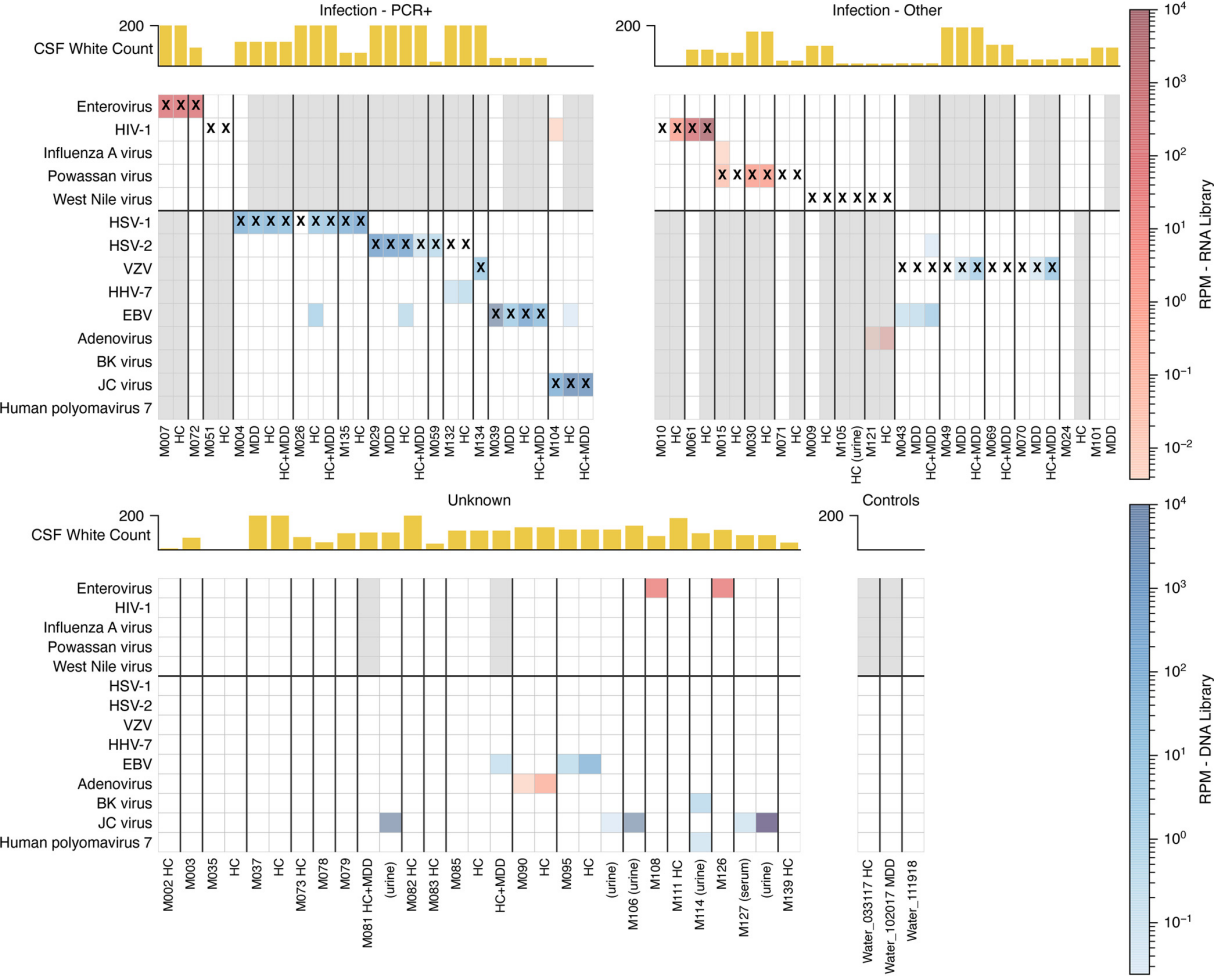
Viral taxa identified in cerebrospinal fluid using mNGS with or without enhanced methods. A heat map shows viral taxa identified in each sample. Rows are viral taxa, and columns are samples, some with enhanced sequencing methods (HC and/or MDD). Only classifications with over 100 unique kmers, with at least 1 BLAST-confirmed read, and manually reviewed as noncontaminants are shown. Rows are grouped by RNA viruses (top section) or DNA viruses (bottom section). The color intensity corresponds to the RPM of the taxa. Red boxes correspond to detection in RNA libraries, while blue boxes correspond to detection in DNA libraries. Some DNA viruses were detected in RNA libraries (e.g., adenovirus in subject M121). Gray-shaded columns represent samples that did not undergo DNA or RNA sequencing. Samples in which a contaminant was found are included here as blank columns, and the contaminants are shown in Fig. S4 in the supplemental material. X’s represent the clinical diagnosis. Yellow bars indicate the CSF nucleated cell count for each subject. The four groupings of columns from the top left to the bottom right correspond to infections diagnosed by positive PCR, infections diagnosed by nonmolecular techniques, subjects with an unknown etiology, and negative controls, including extracted water.

**FIG 4:**
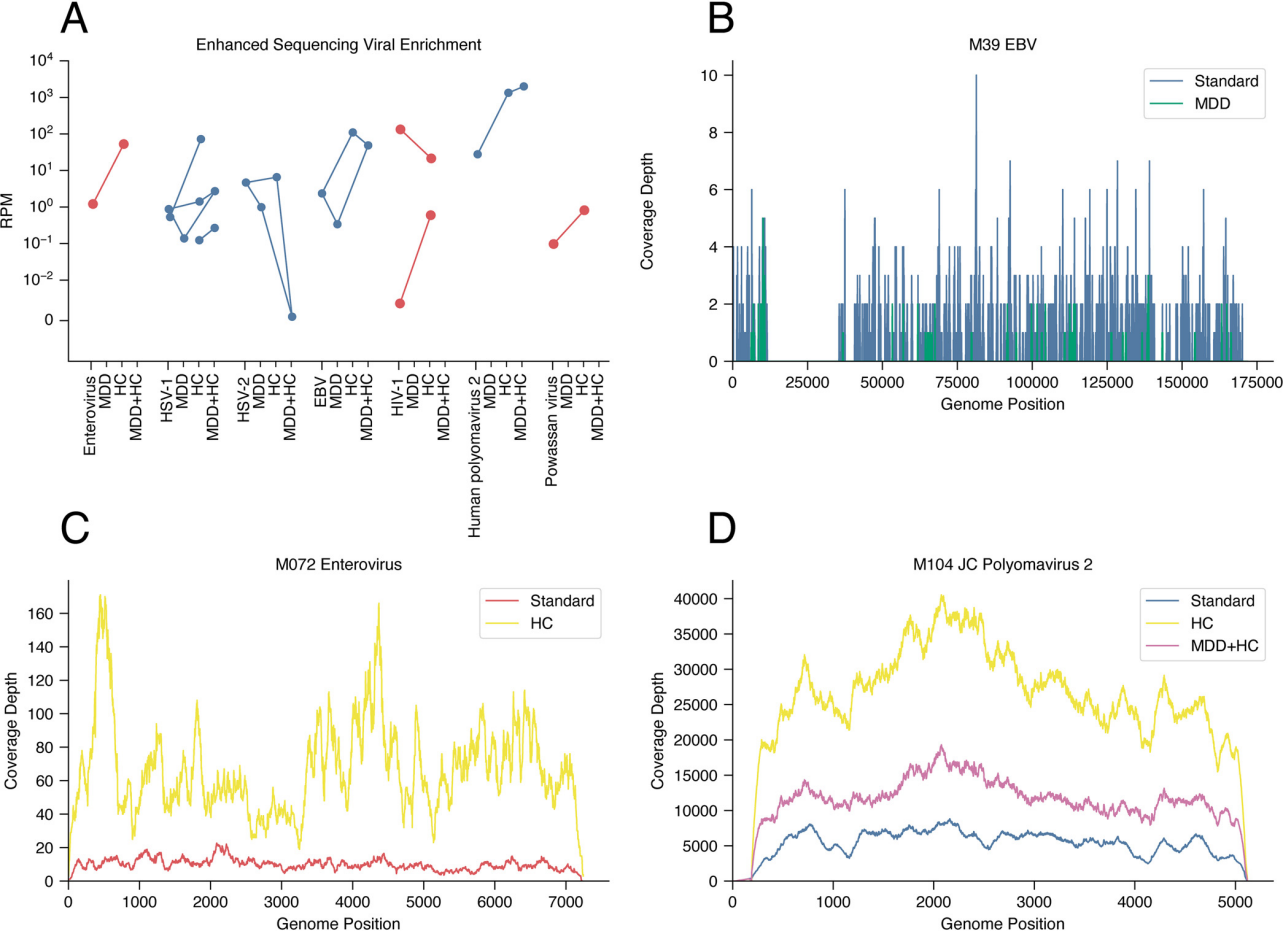
Enhanced methods for mNGS. (A) Comparison of specific viral abundances among the non-computationally depleted reports for HC, MDD, and HC plus MDD for RNA samples (orange) and DNA samples (blue). (B to D) Hybrid capture improved the overall coverage for DNA and RNA viruses such as EBV (B), enterovirus (C), and JC polyomavirus 2 (D). Methylated DNA depletion improved the coverage for some DNA viruses such as JC polyomavirus 2 (D) but not others such as EBV, which utilizes host methylation in its life cycle (B).

To understand mNGS performance in a real-world context, we sequenced 68 CSF, 3 plasma, 5 serum, and 12 urine samples along with 47 negative controls. We performed mNGS from RNA, DNA, or both, generating an average of 9.6 million reads per subject (Fig. S3; see also Tables S4 to S6 at https://figshare.com/articles/dataset/Tables/13266506). We identified a plausible pathogen in 22 subjects (32.4%): 18 by standard mNGS, an additional 2 with the use of HC, and 2 more with the use of MDD (Fig. 3; Fig. S4; see also Table S7 at https://figshare.com/articles/dataset/Tables/13266506). As expected, we detected viral nucleic acid in most subjects in the “infection, CSF PCR^+^” group (10 out of 12 [83%]) (Fig. 3), consistent with other mNGS studies (18, 23). mNGS was negative in one subject with herpes simplex virus 2 (HSV-2) infection and another with human immunodeficiency virus type 1 (HIV-1) infection, illustrating that mNGS can be less sensitive than PCR for very low-level infections (Text S1 and Fig. S5). We detected reads from both JC virus and HIV in a subject with HIV and progressive multifocal leukoencephalopathy (PML), illustrating the capacity of this single platform to identify viral coinfections. In assessing our enhanced methods, we found that HC increased the number of viral reads in 8 out of 9 cases positive by routine mNGS, sometimes substantially (Fig. 4). In contrast, MDD led to mixed results, enabling pathogen detection in 4 out of 19 matched libraries (e.g., varicella-zoster virus [VZV] in subjects M049 and M070) and enhanced yields in 5 additional libraries while decreasing the yields in 10 libraries (e.g., Epstein-Barr virus [EBV] in subject M095) (Fig. 4; Text S1).

10.1128/mBio.01143-21.4 FIG S3 Sequencing metrics for various stages of the computational pipeline. (A) Total number of reads in each sequencing library from raw demultiplexed reads through the stages of quality control/trimming, human depletion, deduplication, and negative depletion. (C) Distribution of the percentage of reads retained after each incremental step for all samples. (B) Comparison of human abundances for each subject between routine, hybrid capture (HC), methylated DNA depletion (MDD), and hybrid capture plus methylated DNA depletion (HC+MDD) analyses of DNA samples. (D) Comparison for RNA. Download FIG S3, SVG file, 0.7 MB. Copyright © 2021 Piantadosi et al. 2021 Piantadosi et al. https://creativecommons.org/licenses/by/4.0/ This content is distributed under the terms of the Creative Commons Attribution 4.0 International license.

10.1128/mBio.01143-21.5 FIG S4 Unfiltered metagenomic classifications, including contaminants. A heat map shows viral taxa identified in each sample type. Compared to Fig. 2, this figure shows all classified taxa without manually screening out contaminants. Rows are viral taxa, and columns are sample types, some with enhanced sequencing methods (HC and/or MDD). Only classifications with over 100 unique kmers and at least 1 BLAST-confirmed read are shown. Rows are grouped by whether they are RNA viruses versus DNA viruses (top versus bottom section). The color intensity corresponds to the RPM of the taxa. Red boxes correspond to detection in RNA libraries, while blue boxes correspond to detection in DNA libraries. Stars represent the clinical diagnosis. Gray-shaded columns represent samples that did not undergo DNA or RNA sequencing. The yellow bars indicate nucleated cell counts in the CSF for each subject. The four groupings of columns from the top left to the bottom right correspond to infections diagnosed by positive PCR, infections diagnosed by nonmolecular techniques, subjects with an unknown etiology, and negative controls, including water. Download FIG S4, SVG file, 0.7 MB. Copyright © 2021 Piantadosi et al. 2021 Piantadosi et al. https://creativecommons.org/licenses/by/4.0/ This content is distributed under the terms of the Creative Commons Attribution 4.0 International license.

10.1128/mBio.01143-21.6 FIG S5 Results of HSV-2- and HIV-1-specific PCRs. (A) Amplification curve analysis demonstrating that CSF from subject M029 (blue) (positive control) amplified in three out of three replicates (mean threshold cycle [*C_T_*] = 23.8), consistent with positive mNGS results for HSV-2. CSF from subject M132 (gray) amplified in only one out of three replicates (*C_T_* = 39.8); correspondingly, no HSV-2 reads were detected by mNGS. There was no amplification from the negative control (red). (B) Melting-curve analysis demonstrating consistent curves across all positive wells. (C) Amplification curve analysis demonstrating that CSF from subject M061 (purple) (positive control) amplified in three out of three replicates (mean *C_T_* = 25.2), consistent with positive mNGS results for HIV-1. CSF from subjects M051 (blue) and M010 (gray) amplified at high *C_T_* values, similar to the negative control (red). (D) Melting-curve analysis demonstrating that only one replicate from subject M051 melted in a pattern consistent with the positive controls. The other positive wells melted at lower temperatures, suggestive of nonspecific amplification or primer dimerization. (E) Gel electrophoresis results from PCR products demonstrating a band of the expected size for subject M061 (positive control) and a faint band of the expected size for subject M051 but not subject M010 or the negative control. Download FIG S5, SVG file, 0.2 MB. Copyright © 2021 Piantadosi et al. 2021 Piantadosi et al. https://creativecommons.org/licenses/by/4.0/ This content is distributed under the terms of the Creative Commons Attribution 4.0 International license.

### mNGS and enhanced methods detect pathogens not traditionally detected by CSF PCR.

Twenty-five subjects in the “infection, other” group had infections diagnosed by serology from CSF and/or blood or by PCR from blood (Table 1). Fifteen had an infection for which no clinically approved CSF PCR assay was available; standard mNGS detected pathogen nucleic acid in six, and mNGS plus HC detected pathogen nucleic acid in a seventh, yielding 7/15 positive hits (47%) (Fig. 3). There were several cases of regional interest. For example, three subjects were clinically diagnosed with Powassan encephalitis using a time-consuming send-out serology test, and mNGS identified Powassan virus RNA in two cases. In addition, while our methods were focused on viral detection, we identified atypical bacteria whose genome reads were readily distinguishable from the background, including Borrelia burgdorferi in two out of two subjects diagnosed with Lyme disease by serology and Anaplasma phagocytophilum in a subject diagnosed by PCR from blood (Fig. S6).

10.1128/mBio.01143-21.7 FIG S6 Detection of atypical bacteria. A heat map shows the recovery of sequencing reads for a subset of atypical bacteria. Only samples classified with over 100 unique kmers and at least 1 BLAST-confirmed read are shown. The color intensity corresponds to RPM in DNA samples. Download FIG S6, SVG file, 0.02 MB. Copyright © 2021 Piantadosi et al. 2021 Piantadosi et al. https://creativecommons.org/licenses/by/4.0/ This content is distributed under the terms of the Creative Commons Attribution 4.0 International license.

In the remaining 10 subjects from the “infection, other” group, clinical CSF PCR was available and negative for the culprit pathogen (human herpesvirus 6 [HHV-6] [*n* = 1], VZV [*n* = 3], West Nile virus [WNV] [*n* = 3], and mycoplasma [*n* = 3]). While all of these were negative using standard mNGS, the addition of MDD allowed the detection of VZV in two subjects (M049 and M070) (Text S1; see also Fig. S7 at https://figshare.com/articles/figure/Supplemental_Figures/13266488). In both cases, clinical CSF VZV PCR from the same sample was negative, illustrating that mNGS may occasionally be more sensitive than a clinically validated PCR. In contrast, MDD decreased the yields for other herpesviruses, suggesting pathogen-specific effects (Fig. 4; Text S1).

### mNGS detects pathogens not tested by clinicians.

Among the 24 subjects with no identified clinical diagnosis (“unknown”), standard mNGS identified viruses in 3 subjects, and no additional pathogens were detected using MDD and HC. We detected enterovirus in two subjects with lymphocytic meningitis (subjects M108 and M126), neither of whom had orders for clinical enterovirus PCR. These findings were verified by sequencing a second CSF aliquot and by assembling a complete enterovirus genome for each subject. Phylogenetic analysis for both subjects demonstrated closely related echovirus 30 strains (see Fig. S8 at https://figshare.com/articles/figure/Supplemental_Figures/13266488).

We also detected EBV and assembled a complete genome in one subject (M095) during two serial hospitalizations for recurrent lymphocytic meningitis. While clinical testing for EBV in CSF was not performed, EBV PCR was positive from blood during both admissions. Overall, these results are compatible with EBV meningitis or reactivation in the setting of another, unidentified primary syndrome (26).

### mNGS and enhanced methods detect viruses of uncertain significance.

In addition to the plausible pathogens described above, we detected DNA viruses of uncertain clinical significance. EBV was present at low levels in CSF from four subjects, three of whom had alternative primary diagnoses: VZV (subject M043), HSV-1 (M026), and HSV-2 (M029). For the fourth subject (M085), no alternative diagnosis was identified; however, EBV reads were detected only after MDD and HC, and a clinical PCR for EBV from CSF was negative. A review of clinical data for these subjects suggested that EBV was unlikely to explain their clinical syndromes, and these findings most likely suggest reactivation in the setting of another acute process.

We also detected human herpesvirus 7 (HHV-7) at a low level in a subject (M132) who was diagnosed with HSV-2 by clinical PCR, but HSV-2 was not detected by mNGS. Acute encephalitis due to HHV-7 rarely occurs in immunocompetent adults and has been described in three cases of patients with limbic encephalitis (27), facial cranial palsy, and polymyeloradiculitis (28, 29); none of these syndromes were compatible with this subject’s presentation. Adenovirus reads were detected in two subjects (M090 and M121) and were not considered vector contaminants due to their distribution across the genome; however, the reads were found in RNA libraries only, and subjects were not known to be immunocompromised or to have features compatible with adenovirus infection.

A known challenge of mNGS is the assessment and interpretation of background contamination. Even after extensive computational depletion of both human reads and sequences found in negative controls, bacteria accounted for ∼11% of DNA and ∼39% of RNA reads. We also found viral reads from bacteriophages and vectors commonly used in molecular biology, such as adenovirus, cytomegalovirus (CMV), HIV/lentiviruses, and parvoviruses, consistent with previous studies (30). Finally, we found a few reads matching recently discovered picornaviruses from environmental surveys (Text S1; see also Table S8 at https://figshare.com/articles/dataset/Tables/13266506) (31).

### mNGS is negative for subjects with noninfectious diagnoses.

mNGS did not detect pathogen nucleic acid in the seven subjects with noninfectious diseases (“alternative diagnosis” group): autoimmune encephalitis and cerebellitis (*n* = 3), lymphoma (*n* = 2), and vasculitis (*n* = 2). In this category, the median CSF white blood cell (WBC) count was 2 to 6 times lower than those in the two infection groups. The “alternative diagnosis” group had the highest number of ID tests ordered for CSF and blood (median, 26 tests [range, 10, 57]) (see Table S2 at https://figshare.com/articles/dataset/Tables/13266506), which is consistent with provider practice to test a wide range of pathogens prior to immunomodulatory therapy; subjects were ultimately treated with immunosuppressive agents.

## DISCUSSION

Advances in genomic technologies provide translational researchers the unprecedented capacity to identify and study pathogens in patients with meningitis and encephalitis. Here, we performed a prospective study using mNGS, enhanced laboratory and analysis techniques, and detailed clinical phenotyping to assess the use of this technology as a diagnostic tool for hospitalized subjects with inflammatory CSF. We identified a range of CNS pathogens, including regionally important tick-borne organisms not typically detected by CSF nucleic acid testing. In 9 cases, we were able to recover full or partial viral genomes, demonstrating the utility of this technique for virus characterization studies (e.g., molecular epidemiology and identification of neurotropic variants). In our study, subjects with CNS infections diagnosed using CSF PCR underwent fewer ID tests than other clinical groups with inflammatory CSF and had shorter lengths of hospital stay (32); from this, we infer that the judicious application of molecular diagnostic techniques such as mNGS can positively impact patient care and associated costs. Together with recent reports (18), this work highlights the opportunity for mNGS to become integrated into the infectious disease diagnostic toolkit.

Overall, mNGS was highly effective at detecting pathogens identified by clinical PCR. mNGS detected the expected pathogen in 10 of 12 subjects, similar to a recent study detecting viruses in 14 out of 16 subjects diagnosed by CSF PCR (18). Our results also highlight the benefit of enhanced mNGS techniques. Panviral HC consistently improved the sequencing of RNA and DNA viruses and resulted in virus detection in two cases (HSV-1 and HIV) that were negative by standard mNGS. MDD plus mNGS detected VZV DNA in two samples negative by standard mNGS. However, MDD decreased the yields for some viruses, indicating that the role of this technique in mNGS remains unclear. Our mixed results with MDD correlate with previous studies focusing on bacterial metagenomics (33, 34). Saponin lysis of host cells may prove a more effective depletion technique for DNA (35, 36).

A notable strength of this study was the detection of pathogens not routinely detected by CSF PCR, most notably the tick-borne pathogens Powassan virus (17), Borrelia burgdorferi, and Anaplasma phagocytophilum. These pathogens show increasing rates of human infection (37), particularly in the Northeastern United States, where this study was conducted. For Powassan virus, which is routinely detected by serology, our findings illustrate the potential utility of nucleic acid-based screening. Interestingly, we detected the CSF presence of *Anaplasma*, which is not commonly considered to be a cause of CNS infection (38), although the related intracellular bacterium Ehrlichia chaffeensis can cause meningoencephalitis (39, 40). Overall, the high number of subjects with tick-borne infection highlights the importance of conducting mNGS in diverse geographical regions for both diagnostic purposes and epidemiological studies.

Among the 24 subjects for whom no diagnosis was achieved by routine clinical testing (“unknown”), mNGS detected potential pathogens in 3 (8%), a rate similar to that reported previously by Wilson et al. (13/159 [8%]) (18). It is possible that subjects in whom no pathogen nucleic acid was detected had a noninfectious syndrome or an infection with a low pathogen burden or short duration of replication. We reviewed the postdischarge clinical course in the subgroup, and none were identified as having an infectious syndrome, signaling the likelihood that mNGS did not miss an actionable result.

Our results highlight a few challenges associated with mNGS, particularly for infections with low titers or parainfectious complications. For example, we report an equivocal mNGS result in a subject with HIV-1 who had a CSF HIV load of 469 copies/ml, a value close to the recently reported CSF limit of detection of 313 copies/ml for HIV-1 using mNGS (23). Additionally, mNGS results were negative in all four subjects with WNV, three of whom had clinical WNV PCRs from CSF performed, which were also negative. These results support other studies showing that WNV nucleic acid is usually undetectable in CSF by clinical PCR (23, 41) or mNGS (18), although it may be observed in immunocompromised subjects (15, 41–44). Similarly, CSF mycoplasma nucleic acid was not detected clinically or by mNGS in three subjects despite positive mycoplasma IgM serologies. These patients had clinical and neuroradiographic findings suggestive of encephalitis or encephalomyelitis, including multifocal T2/FLAIR (T2-weighted fluid-attenuated inversion recovery) hyperintensities in the brain and spinal cord (M006), superrefractory epilepsy with T2/FLAIR hyperintensity in the right posterior subinsular/anterior temporal stem (M032), and longitudinally extensive transverse myelitis (M075). This finding supports interpretations that CNS complications of mycoplasma infections likely reflect a parainfectious antibody-mediated response rather than direct infection (45).

While we investigated specific atypical bacteria of interest (*Borrelia*, *Anaplasma*, and *Mycoplasma* spp.), our study focused on viruses for four key reasons: they are the most common pathogens in CNS infection (4, 5, 24, 25); bacteria and fungi often require different laboratory methods for processing and nucleic acid extraction (34); bacterial infections are associated with greater pleocytosis and, therefore, higher levels of host background (23); and the analysis of viruses is more tractable given that mNGS (34) commonly detects bacterial reads (e.g., Pseudomonas aeruginosa and Escherichia coli) as background from skin and reagents (34). As this was not a clinical validation study, we focused on the practical application of mNGS in a defined cohort rather than general diagnostic test performance (21, 23). We adhered to strict practices to minimize contamination, but we did not conduct this research study in a Clinical Laboratory Improvement Amendments (CLIA)-certified laboratory (22), allowing us flexibility in iterative testing and refinement of methods. Because this study was conducted primarily using clinical excess samples, many of which had undergone multiple prior freeze-thaw cycles for clinical testing, it is also possible that some infections were missed due to nucleic acid degradation prior to mNGS, which would be solved if clinical processing for mNGS is standardized (34).

### Conclusions.

Overall, our results highlight several important benefits of mNGS, including opportunities to reduce dependence on test-specific diagnostics, recover pathogen genomic data, and potentially offer shorter turnaround times than serology (17). However, our results among subjects with an unknown etiology of disease suggest that the addition of mNGS to standard clinical testing will lead to relatively few additional diagnoses, underscoring the challenge of identifying an etiology in these devastating clinical syndromes. One potential strategy for incorporating mNGS into clinical diagnostic workflows would be wide implementation early in the diagnostic workup to capitalize on the one-step detection of common pathogens, potentially sparing subjects unnecessary tests and reducing overall costs. An alternative would be to reserve this specialized technique for subjects with a high pretest probability of infection (e.g., immunocompromised). Determining how to best utilize mNGS in clinical practice will require evaluation of these factors as well as the cost and logistics of implementation (46, 47). Currently, it is prudent to employ diagnostic mNGS through close communication between clinicians and mNGS experts (18) to evaluate the plausibility of the pathogens identified. This is especially important considering background reads and contamination, the essential limitation that mNGS detects only infections with circulating pathogen nucleic acid, and our still-evolving understanding of mNGS test characteristics. Results from this study will inform ongoing efforts to transition the much-needed and promising technique of mNGS from a research tool to a clinical test used in the routine care of patients with suspected CNS infection.

## MATERIALS AND METHODS

### Subject enrollment and clinical characterization.

The Prospective Encephalitis and Meningitis Study (PEMS) is a prospective cohort study enrolling adults who present to Massachusetts General Hospital (MGH) with confirmed or suspected CNS infection. Adults aged ≥18 years with at least one of the following symptoms were eligible for enrollment: (i) altered level of consciousness, (ii) fever, (iii) seizure, (iv) focal neurological findings, (v) electroencephalographic or neuroimaging findings consistent with encephalitis or meningitis, and (vi) refractory headaches. Additionally, enrollment was offered only to patients who had undergone, or planned to undergo, lumbar puncture (LP). Potential participants were referred to the study team by clinicians concerned for encephalitis or meningitis. In parallel, the study team performed queries of the electronic medical record to screen for eligible participants based on the reason for admission and/or CSF results. Further details of screening and enrollment are provided in Text S1 in the supplemental material.

A total of 136 subjects were prospectively enrolled in the PEMS between April 2016 and December 2017, of whom 122 had available CSF for mNGS. For this study, immunocompetent patients with a CSF white blood cell count (WBC) of <5 cells/μl (*n* = 40) were excluded as being unlikely to have infectious meningitis or encephalitis; most of these patients had been enrolled prior to undergoing LP (Text S1). Patients with infection due to nosocomial bacteria, or bacteria and fungi that would be challenging to distinguish from common laboratory contamination in mNGS, were also excluded (*n* = 14) (Table S1). Sixty-eight subjects were ultimately included in the mNGS analysis (Fig. 1). This study was approved by the Partners Institutional Review Board under protocol 2015P001388. Further details are in Text S1.

10.1128/mBio.01143-21.8 TABLE S1 Subjects not meeting inclusion criteria due to known bacterial or fungal infection. Cerebrospinal fluid (CSF) total nucleated cells (TNC) and pathogen classification for the 14 cases excluded from analyses are shown. Download Table S1, XLSX file, 0.00 MB. Copyright © 2021 Piantadosi et al. 2021 Piantadosi et al. https://creativecommons.org/licenses/by/4.0/ This content is distributed under the terms of the Creative Commons Attribution 4.0 International license.

### Nucleic acid isolation and standard mNGS.

To minimize environmental contamination from viruses studied in the research laboratory, nucleic acid extraction and library construction were performed in an isolated workspace with limited access, extensive decontamination, and strict oversight of supplies, storage areas, and reagents. As a negative control, water and/or CSF from an uninfected patient (negative CSF) was included with each batch starting from nucleic acid isolation. Nucleic acid was extracted from 140 μl of CSF, urine, or plasma stabilized with linear acrylamide using the QIAamp viral RNA minikit (Qiagen). The eluent was split into two fractions for RNA and DNA sequencing. External RNA Controls Consortium (ERCC) spike-in oligonucleotides were added to each fraction; for the RNA fraction, RNA spike-in oligonucleotides were synthesized according to National Institute of Standards and Technology instructions, and for the DNA fraction, cDNA spike-in oligonucleotides were synthesized from RNA templates using random hexamer primers (48). Samples also underwent cDNA synthesis using random hexamer primers and previously described methods (49, 50). Both DNA and RNA libraries underwent tagmentation with the Nextera XT DNA library prep kit (Illumina) and were pooled and sequenced on HiSeq and MiSeq machines using paired-end 100- or 150-bp reads. Methods are outlined in Fig. 1B, and details are in Text S1.

### Methods to enhance the detection of pathogen nucleic acid.

We attempted two different approaches to enhance sequencing-based detection of pathogen nucleic acid, either separately or together. We first assessed whether enrichment for nonmethylated microbial DNA would improve mNGS yields. We used samples from 12 subjects: 10 with clinically diagnosed DNA virus infections and 2 with clinically diagnosed Lyme disease. Samples underwent methylated DNA depletion (MDD) using the NEBNext microbiome DNA enrichment kit (New England BioLabs), and the enriched fraction was used for DNA library construction as described above (Fig. 4). This kit is designed to remove human host DNA that is methylated and is expected to enrich viral content because no known viral pathogens encode a methyltransferase. We then assessed the efficacy of enrichment for viral nucleic acid by hybrid capture (HC) using a set of probes targeting all viruses known to infect humans (51). We applied HC to 13 RNA and 12 DNA libraries from subjects with clinically diagnosed RNA and DNA virus infections, respectively. Given the observed efficacy of HC, we also applied HC to samples from 20 subjects in the “unknown” group (using the RNA library, DNA library, or both depending on clinical suspicion for a specific pathogen). To perform HC, indexed libraries were pooled into groups of approximately 5 samples per reaction and then underwent hybridization and capture using the SeqCap EZ enrichment kit (Roche), with modifications as described previously (51). HC libraries were pooled and sequenced as described above. Finally, we applied both MDD and HC to a subset of 12 samples from patients with known or suspected DNA virus infection.

### Metagenomic analysis pipeline.

Illumina sequencing reads were demultiplexed via viral-ngs (57), quality filtered and read trimmed using Trimmomatic (52), and depleted of human reads via a comprehensive KrakenUniq (53) database. The resulting reads were deduplicated and assembled into metagenomic contigs via metaSPAdes (54). Contigs were classified using a cascading BLAST scheme in which unclassified contigs at each stage passed to the next level of more intensive BLAST searches from MegaBLAST and BLASTn to BLASTx (55, 56). Contigs and associated hits derived from water and negative-control samples were aggregated into a contaminant database and used to further deplete the human-depleted reads (Fig. 1C; Fig. S1).

10.1128/mBio.01143-21.2 FIG S1 Computational processing workflow. (A) Sequencing reads first underwent universal quality control, human depletion (via stringent criteria of >20% kmers within the read classifying specifically to a human taxid), and deduplication. (B) These reads were assembled into contigs, and >600-bp contigs were subjected to BLAST analysis to recover strong reference matches for long contigs. These were used as a “negative-control” depletion database, after which the remaining reads were classified via the comprehensive KrakenUniq and Kaiju databases. Viral hits were validated using BLASTn. Download FIG S1, SVG file, 0.01 MB. Copyright © 2021 Piantadosi et al. 2021 Piantadosi et al. https://creativecommons.org/licenses/by/4.0/ This content is distributed under the terms of the Creative Commons Attribution 4.0 International license.

Finally, the human- and contaminant-depleted reads (see Table S4 at https://figshare.com/articles/dataset/Tables/13266506) were classified by KrakenUniq using the same comprehensive database as the one described above. Reads classified as potentially human-pathogenic viruses were validated via BLAST, discarding any reads that were not concordantly classified by both methods. The counts of reads per taxon were normalized to sequencing depth as reads per million (RPM) (see Tables S5 and S6 at https://figshare.com/articles/dataset/Tables/13266506). Kaiju (58) was run on depleted reads to explore divergent taxon hits, while viral-ngs was used to assemble genomes for a subset of viruses. For mNGS interpretation after computational classification, false-positive species were identified based on broad contamination patterns across all sequencing runs. All true-positive determinations were output from the mNGS classification results directly.

### Statistical analyses.

Analyses were performed using Student’s *t* test and the Mann-Whitney U test for normally and nonnormally distributed continuous variables, respectively, and using the χ^2^ test for categorical variables.

### Data availability.

Reads after quality control (QC) filtering, trimming, and depletion of human reads via KrakenUniq to a comprehensive database, including the human genome (GRChg38/hg38) and all human sequences from the BLAST NT database, are available in the NCBI Sequence Read Archive (SRA) under accession number PRJNA668392.
