# Supplementary material for: Enhanced Virus Detection and Metagenomic Sequencing in Patients with Meningitis and Encephalitis
Source: mBio. 2021 Aug 31;12(4):e01143-21. doi: 10.1128/mBio.01143-21 (PMC8406231; doi:10.1128/mBio.01143-21)
Supplement: TEXT S1 [file mbio.01143-21-s0001.docx]

**Supplementary Material**

**Enhanced virus detection and metagenomic sequencing in patients with meningitis and encephalitis**

**Authors:**

Anne Piantadosi^a,b,c#^*, Shibani S. Mukerji^d,e^*, Simon Ye^a^*, Michael J. Leone^d^, Lisa M. Freimark^a^, Daniel Park^a^, Gordon Adams^a,b^, Jacob Lemieux^a,b,e^, Sanjat Kanjilal^b,e,f^, Isaac H. Solomon^e,f^, Asim A. Ahmed^e,g^, Robert Goldstein^b,e^, Vijay Ganesh^a,e,h^, Bridget Ostrem^d,e^, Kaelyn C. Cummins^i,ɑ^, Jesse M. Thon^d,e,β^, Cormac M. Kinsella^a,m,γ^, Eric Rosenberg^b,e,j^, Matthew P. Frosch^d,e,j^, Marcia B. Goldberg^b,e^, Tracey A. Cho^d,e,k+^, Pardis Sabeti^a,b,e,l,m,n+^

*Equal contribution

Anne Piantadosi, Shibani S. Mukerji and Simon Ye all contributed equally to this work. Author order was determined by mutual agreement among first authors.

^+^ Equal contribution

**Affiliations:**

^a^Broad Institute of MIT and Harvard, Cambridge, MA

^b^Division of Infectious Diseases, Massachusetts General Hospital, Boston, MA

^c^Emory University School of Medicine, Atlanta, GA

^d^Department of Neurology, Massachusetts General Hospital, Boston, MA

^e^Harvard Medical School, Boston, MA

^f^Department of Pathology, Brigham and Women’s Hospital, Boston, MA

^g^Department of Pediatrics, Harvard Medical School, Children’s Hospital, Boston, Massachusetts

^h^Department of Neurology, Brigham and Women’s Hospital, Boston, MA

^i^Division of Infectious Diseases, Brigham and Women’s Hospital, Boston, MA

^j^Department of Pathology, Massachusetts General Hospital, Boston, MA

^k^University of Iowa, Department of Neurology, Iowa City, IA

^l^Department of Organismic and Evolutionary Biology, Harvard University, Cambridge, MA

^m^Department of Immunology and Infectious Disease, Harvard T.H.Chan School of Public Health, Boston, MA

^n^Howard Hughes Medical Institute, Chevy Chase, MD

Current affiliates:

^ɑ^Baylor College of Medicine, Houston, TX

^β^Department of Neurology, University of Pennsylvania, Philadelphia, PA

^γ^Amsterdam UMC, Amsterdam, The Netherlands

**Supplementary Methods:**

*Participant enrollment and criteria for analysis*

The Prospective Encephalitis and Meningitis Study (PEMS) enrolled adults ages ≥18 years who presented to Massachusetts General Hospital (MGH) with confirmed or suspected CNS infection. Information about the study was directed to hospital providers most likely to perform lumbar punctures and included presentations by the study team and distribution of flyers, cards and emails; a dedicated email address for the study was created. Patients were referred to the study team by practitioners in Neurology, Infectious Disease and Internal Medicine when planning or had completed a lumbar puncture. Once a potential participant was identified, the study coordinator reviewed the record to ensure the patient met inclusion criteria: 1) altered level of consciousness; 2) fever; 3) seizure; 4) focal neurological finding; 5) electroencephalographic or neuroimaging findings consistent with encephalitis or meningitis; and 6) refractory headaches.

In parallel, the study team used queries within the institution’s electronic health record to allow the study coordinator and Principal Investigators of PEMS to identify potential study participants in the timeliest manner. Search terms included orders for CSF herpes simplex virus or enterovirus PCR as these tests are commonly performed in patients with encephalitis or meningitis, and reasons for admission to the Neurology intensive care unit or floor given that majority of patients with suspected CNS infection and who did not receive a LP in the emergency department would be admitted to the service. In these cases, the study coordinator contacted a member of the primary team to request permission to approach the patient. If granted, the study coordinator contacted the patient or proxy with information about the study and the option of participating.

Immunocompetent patients with CSF white blood cell count (WBC) < 5 cells/ul (n = 40) were excluded as unlikely to have active CSF infection. Of the 40 excluded immunocompetent participants, 33 were consented prior to LP (17 HIV-1 infected on virally suppressive antiretroviral therapy (ART)). The remaining 7 out of 40 participants had normal CSF white blood cell counts and while consented into the study given an initial differential diagnosis that included possible infection, these subjects were deemed unlikely to have active CSF infection following clinical expert review by an Infectious Disease or Neurology trained clinician.

This study was approved by the Partners Institutional Review Board under protocol 2015P001388, and subjects signed a written statement of informed consent. The Broad Institute of MIT and Harvard has a standing reliance agreement with Partners through which it relied on Partners IRB to provide a review of this study.

*Study Definitions*

Data were extracted from the medical record and reviewed by at least one clinical reviewer not involved in the subject’s care who had clinical training in Infectious Disease (A.P., M.G., R.G) or Neurology (T.C., S.M.). Subjects diagnosed with a viral infection by clinical PCR testing from CSF were classified as “Infection, CSF PCR+”, subjects diagnosed with infection by another method such as serology or PCR from blood were classified as “Infection, Other”, subjects in whom clinical diagnosis was definitively determined to be of an etiology other than infection were classified as “Alternative Diagnosis”, and subjects with no etiology identified up to one month post-discharge were classified as “Unknown.” Length of stay (LOS) was determined from admission to discharge at MGH. The number of distinct diagnostic ID tests ordered for each subject was gathered from the electronic medical record, and calculated from hospital admission to discharge. Testing results from the Emergency Department, outpatient clinics or a transferring institution were excluded. We also excluded tests cancelled by the treatment team or credited as possible duplicates.

The time from symptom onset to lumbar puncture (LP) was calculated for each subject and classified as acute (0-3 days), early subacute (4-7 days), late subacute (8-30 days), or chronic (> 30 days) (Table 1). Based on chart review, subjects were identified as immunocompromised if they met any of the following criteria: living with AIDS (CD4 < 200), leukemia, systemic lymphoma or other hematological malignancy, severe combined immunodeficiency, or use of cytotoxic or immunosuppressive agents that include chemotherapy, rituximab, cyclophosphamide, azathioprine, TNF-alpha inhibitors, monoclonal antibodies and corticosteroids at dosages equivalent to or greater than prednisone 20 mg/d for past 30 days. Details of the clinical presentation were abstracted from chart review, and the presence of photophobia, neck stiffness or altered mental status was classified as ‘yes’ if its presence was marked at least twice in the medical record by different providers.

*PCR Validation*

In two subjects, clinical testing identified a virus that was not detected by mNGS, and we performed confirmatory PCR using virus-specific assays. HSV-2 PCR was performed using the Power SYBR Green PCR Master Mix (Applied Biosystems) with 10µM primers (fwd: TACGCTCTCGTAAATGCTTC, rev: GCCCACCTCTACCCACAA) in a 10µl assay including 3µl of template. PCR conditions were as follows: 95°C for 10 minutes; 45 cycles of 95°C for 15 seconds and 60°C for 30 seconds; and 95°C, 55°C, and 95°C for 15 seconds each. HIV-1 RT-PCR was performed using the QuantiFast SYBR Green RT-PCR kit (Qiagen) with 10µM primers (SK145 fwd AGTGGGGGGACATCAAGCAGCCATGCAAAT, SK431 rev TGCTATGTCACTTCCCCTTGGTTCTCT) in a 20µl assay with 4µl template. PCR conditions were as follows: 50°C for 10 minutes; 95°C for 5 minutes; 40 rounds of amplification at 95C for 10 seconds and 60C for 30 seconds; and 95°C, 60°C, and 95°C for 15 seconds each. Due to the observation of primer-dimer background in this assay, PCR products were run on a 2.2% Agarose FlashGel (Lonza) to confirm the presence of an amplicon of the expected size.

*Nucleic acid isolation and standard mNGS*

For RNA mNGS, the RNA fraction was treated with HL dsDNAse (ArcticZymes) per the manufacturer’s instructions. An oligonucleotide of known sequence (ERCC) was added to the sample for quality control. RNA library construction methods have been previously described [(1, 2)](https://paperpile.com/c/aJnfHX/978Nz+gO7E8) and are outlined in Figure 1. Briefly, cDNA was constructed using random hexamer primers and Superscript III (Invitrogen) for first-strand synthesis and NEB reagents for second strand synthesis. Sequencing libraries were generated using the Nextera XT DNA Library Prep Kit (Illumina) with dual index pairs that had not been previously used in the research laboratory in order to minimize environmental contamination. Indexing primers were non-biotinylated in order to allow hybrid capture, as described below. Libraries underwent amplification with the following conditions: i) 72°C for three minutes and 95°C for 30 seconds, ii) up to 18 cycles of 95°C for 10 seconds, 55°C 30 seconds, and 72°C for 30 seconds, iii) 72°C for 5 minutes. The libraries were quantified using the KAPA universal complete kit (Roche), pooled to equal concentration, and sequenced on an Illumina platform using paired-end 100bp or 150bp reads. For DNA mNGS, the DNA fraction was treated with Epicentre RNase I (Lucigen) per the manufacturer’s instructions. DNA was quantified and diluted to 0.33ng/µl for input into NexteraXT DNA Library Prep Kit, and libraries were constructed and sequenced as above (Figure 1).

*Metagenomic Data Filtering and Curation*

The raw sequencing data was demultiplexed using the illumina_demux tool of the viral-ngs v1.23.0 package with default parameters. Demultiplexed raw reads were quality filtered and trimmed using trimmomatic v0.38.1 [(3)](https://paperpile.com/c/aJnfHX/FPa3s) using the following command line parameters: ILLUMINACLIP:NexteraPE-PE.fa:2:30:10 LEADING:20 TRAILING:20 SLIDINGWINDOW:4:20 MINLEN:36. The adapter database for the ILLUMINACLIP step consists of the default adapters from the NexteraPE-PE trimmomatic set. Due to the difficulty of handling orphaned single-ended reads after trimmomatic processing, these reads were discarded from further analysis. After read trimming and quality filtering, the reads were conservatively depleted of human reads using a comprehensive KrakenUniq v0.5.7 [(4)](https://paperpile.com/c/aJnfHX/91u7i) database (available publicly on Google Cloud Storage as a pre-built database, gs://sabeti-public-dbs/krakenuniq/krakenuniq.full.20190626.tar.zst, and input fasta sequences, gs://sabeti-public-dbs/krakenuniq/krakenuniq.full.library.20190626.tar.zst) consisting of RefSeq complete genomes for Archaea, Bacteria, Fungi, Plasmids, Protozoa, Viruses, the human reference genome *hg38*~~,~~ the EmVec and UniVec databases, viral neighbors as defined by krakenuniq-download, viral sequences with a human host from the CATCH v1.3.0 [(5)](https://paperpile.com/c/aJnfHX/r9Arq) database (sequences from the NCBI Viral Genomes resource [(6)](https://paperpile.com/c/aJnfHX/rHHiJ)), as well as all sequences in BLAST nt with a taxid of 9606 (*Homo sapiens*) downloaded on 06/26/2019 (available at gs://pathogen-public-dbs/encephalitis/nt.2019.06.26.tar.zst). Since the goal was to conservatively deplete only definitively human reads, only reads classifying exclusively to the human taxon ID with a score greater than 0.2 were removed from further analysis. By using a comprehensive database, reads that match to both human and a microbe are classified at a higher common LCA taxon, and are not depleted by this approach. The conservatively depleted reads were then deduplicated using the *de novo* deduplication tool clumpify.sh from the BBtools package v38.71 [(7)](https://paperpile.com/c/aJnfHX/CnYMm). The parameters used were dedupe=3 passes=3. By default, clusters of reads with edit-distance <=2 were considered part of a single cluster, and only the read with the highest quality score of each cluster was retained. This step was performed to improve abundance estimation and save computational time, with the assumption that highly similar sequences were the result of PCR duplication.

*Metagenomic Classification and Assembly*

The full pipeline is depicted as a flowchart in Supplementary Figure 1. Deduplicated reads underwent several analyses to ensure comprehensive metagenomic classification. First, each sample was directly classified using Kaiju v1.6.3 [(8)](https://paperpile.com/c/aJnfHX/XR9to) using the *nr* database from 03/05/2019 (available as a pre-built database, in the bucket directory gs://sabeti-public-dbs/encephalitis/kaiju/, from fasta file gs://sabeti-public-dbs/encephalitis/blast/v5/nr/nr.fasta.zst) and with KrakenUniq using the same comprehensive database described above for human depletion. The classified KrakenUniq reports were analyzed at the species taxonomic level. For each biological sample, all KrakenUniq reports from replicate sequencing libraries were merged along with their estimated unique k-mer content using a customized version of KrakenUniq. The taxa in the merged KrakenUniq reports with <100 unique kmers (subsequences of length 31) were filtered out from further consideration.

Reads also underwent *de novo* contig assembly using metaSPAdes v3.13.0 [(9)](https://paperpile.com/c/aJnfHX/oDfMz). The resulting contigs were processed using dustmasker 1.0.0. Only contigs with a total of >100 bp of unmasked sequence were retained, with any spans between two masked regions only counted if they were >100bp long. Remaining contigs were classified using a series of cascading BLAST v2.9.0+ [(10)](https://paperpile.com/c/aJnfHX/CooFH) steps. MegaBLAST, BLASTn (each using the *nt* database from 06/26/2016, see link above), and BLASTx using the *nt* or *nr* databases respectively were run on contigs, with each contig only proceeding to the next processing step if inadequately classified at the prior stage. Contigs whose combined BLAST hits with sequence identity >=65% covered less than 30% of the query sequence were considered inadequately classified.

To better filter out reads associated with common background contaminants found in the negative CSF and water controls, assembled contigs >600 bp were separately compiled from the DNA and RNA controls (available as fasta files under gs://sabeti-public-dbs/encephalitis/neg). The strong MegaBLAST and BLASTn hits with a minimum bit score >=70, maximum E-value of 1e-6, minimum identity >90%, and scoring within 10% of the bit score of the top BLAST hit were compiled. These hits’ accession IDs were used to extract sequences from the BLAST *nt* database (downloaded 06/26/2019, see link above) using blastdbcmd to create an expanded set of negative control reference sequences, comprising 67,824 RNA sequences and 11,721 DNA sequences (available under gs://pathogen-public-dbs/encephalitis/neg). These sequences, combined with the *de novo* contigs, were indexed by KrakenUniq for further sequence depletion. The deduplicated reads were classified using KrakenUniq to their corresponding negative control DNA or RNA databases respectively, with reads having a KrakenUniq score of >50 matching anything in the database being depleted and removed from further analysis.

Reads remaining after the second depletion stage were re-classified and analyzed using KrakenUniq and Kaiju as above. These reads are a strict subset of the ones analyzed after the de-duplication step, with the aim of improving classification precision at the cost of increased computational time to incorporate sequences from the negative controls.

*Review of positive mNGS results*

Given the extremely high sensitivity of mNGS, viral reads that were detected by the above analysis pipeline were manually reviewed to assess whether they could represent contamination from other samples on the same sequencing run (“run contaminant”) or from the general laboratory background (“lab contaminant”) [(11, 12)](https://paperpile.com/c/aJnfHX/sHuQW+WFPop). Viral reads were excluded if the same virus was observed in a negative control or multiple other samples that had been run on the same flowcell, or if the reads matched a known virus from another subject or from a strain commonly used in the laboratory (Supplementary Table 4 https://figshare.com/articles/dataset/Tables/13266506). Reads corresponding to viral taxa not normally found in humans, such as *Cucumber green mottle mosaic virus* and phages that affect common contaminant bacteria in the *Pseudomonas* and *Burkholderia* genera, were presumed to be contamination of either the sample or the relevant reference databases. Classified taxa with a low number of assigned reads (typically <100) were evaluated manually via Geneious alignments to reference genomes. Reads whose alignment locations were biased towards specific locations on the genome, or had significant deviations from the consensus reference, were manually filtered out as false-positive hits.

Contamination is an important complication in metagenomic sequence classification, particularly when analyzing low nucleic acid sample types such as CSF. Apart from human nucleic acids, healthy CSF is typically considered sterile in terms of the microbiome. However, given the very high sensitivity of mNGS, microbial reads are frequently detected from skin, reagents, and other microbes studied in the laboratory environment [(13–16)](https://paperpile.com/c/aJnfHX/OhScG+Y8LJS+fjbtg+Um4N7). Distinguishing signal from noise is an essential challenge in mNGS, and our methods offer a mechanism to systematically incorporate sequencing data from negative controls including both “ultrapure” water and excess CSF from hydrocephalus subjects (a non-infectious condition).

### Supplementary Results:

*Clinical Characteristics and Laboratory Parameters of Study Cohort*

Of the 68 adults enrolled, New England was the primary residence for 67, with 81% of subjects (55/68) reporting residing in Massachusetts, 10% (7/68) in New Hampshire, 6% (4/68) in Maine and 1 in Vermont. The two primary hospital services used for subject enrollment were Neurology and Medicine services (54% and 31%, respectively; Table 1).

To understand the burden of clinical testing in subjects with suspected meningitis or encephalitis, we calculated the number of infectious disease (ID) tests ordered, and compared this to the subjects’ length of stay (LOS) (Table 1). The “Infection, CSF PCR+” group had the lowest median number of ID tests (12 [IQR:6,56]) and shortest LOS (4·5 [2,51]). Specifically, compared to the “Infection, other” group, the median number of ID tests in the “Infection, CSF PCR+” group was lower (12·5 vs. 25, p < 0·05) and LOS was shorter but not statistically significant (4·5 vs. 9 days, p = 0·12; Table 1). This suggests that CSF PCR platforms may reduce the burden of clinical testing and could impact LOS. There was no difference between the number of ID tests ordered in the “Infection, Other” compared to the “Unknown” group (25 [IQR: 6,62] vs. 23 [IQR: 6,48], p= 0·23). Not surprisingly, LOS moderately correlated with the number of total ID tests ordered (Spearman’s 𝜌 = 0·65, p<0·01; Figure 2B), as well as with the number of tests ordered from CSF only (Spearman’s 𝜌 = 0·46, p<0·01; Supplementary Figure 2B), and with the number of PCR tests (Spearman’s 𝜌 = 0·46, p<0·01; Supplementary Figure 2C). There was no difference in LOS between clinical diagnostic groups or when normalizing the number of ID tests by LOS (Supplementary Figure 2B and C).

While it would be helpful to predict which patients are likely to have an infection that can be identified by molecular testing, we did not detect clinical features associated with any diagnostic groups. For example, we hypothesized that patients with acute hospital presentations (i.e. shortest time between symptom onset and lumbar puncture) would have a high frequency of infections diagnosed by aCSF PCR assay and thus would be a high-yield target population for mNGS. However, the “Infection, CSF PCR+” group had only two patients (17%; 2/12) with acute (≤ 3 days) presentations, and both were diagnosed with enterovirus (EV) meningitis. The remaining patients had subacute presentations (67%; herpes simplex virus type 1 (HSV-1) (n=3), herpes simplex virus type 2 (HSV-2) (n=3), varicella zoster virus (VZV), Human immunodeficiency virus (HIV)), or chronic presentations with evidence of latent virus reactivation (17%; 1 non-HIV-related Epstein-Barr virus (EBV) (brainstem lymphoma), 1 John Cunningham Virus (JC virus); Supplementary Table 3). In contrast, the “Infection, other” group had 7 out of 25 subjects (28%) with acute presentations, including three arthropod-borne infections (Jamestown Canyon virus, Powassan virus, Lyme disease), VZV, human herpesvirus 6 (HHV-6), mycoplasma, and metapneumovirus. Seventeen subjects (68%) had subacute presentations (VZV (n=5), West Nile virus (WNV) (n=4), mycoplasma (n=2), Powassan virus (n=2), HIV-1 (n=2), Lyme disease, and anaplasmosis), and one subject had a chronic presentation (VZV). In the case of HHV-6, the participant underwent a matched unrelated donor stem cell transplant 15 days prior and was admitted with fever and disorientation. The individual had plasma HHV6 viral load of 16,500 DNA copies/ml (VIRACOR-IBT labs), and elevated CSF white blood cells 9 cells/ul (70% lymphocytes, 29% monocytes), and protein 151 mg/dL. Given that the onset of neurological symptoms in HHV6 encephalitis can be variable and this presentation is temporally earlier than the average presentation, it is possible that CSF viral DNA could have been detected at a later time point. These data, in aggregate, suggest that symptom onset is an unreliable indicator of when molecular versus serology assays should be used, and will be a poor metric for when mNGS should be deployed.

We also did not observe reliable differences in CSF parameters between groups (Table 1). For example, subjects in the “Infection, CSF PCR+” group had non-statistically-significant higher WBC counts in CSF (median 106 cells/uL [36, 377]) when compared to subjects in the “Infection, Other” group (median 47 cells/uL (IQR [14, 105]) and subjects in the “Alternative diagnosis” group (median 17 cells/uL (IQR [10, 25]). However, CSF WBC levels were similar to subjects in the “Unknown” group (median 98 cells/uL) (Supplementary Table 3). Overall these results, suggesting that mNGS integration will be unlikely to depend on timing of symptom onset or basic CSF counts.

*Reduction of human reads by enhanced mNGS*

Whether or not a pathogen was detected, enhanced sequencing techniques were successful in reducing the fraction of human reads. For conventional mNGS samples, human content accounted for ~92% of DNA and ~30% of RNA reads on average. HC reduced the human fraction by 70% and 55% in DNA and RNA samples respectively, while MDD reduced the human fraction in DNA samples by 90%. When combined, these methods reduced the human fraction in DNA by 91%. Thus, both HC and MDD were effective in enriching for non-human content.

*Pathogens missed by mNGS*

mNGS was negative in one subject with HSV-2 (M132) and another subject with HIV-1 (M051), even with the use of HC and high sequencing depth (over 1 million depleted and deduplicated reads in each sample) (Supplementary Table 4 https://figshare.com/articles/dataset/Tables/13266506). Confirmatory PCR assays confirmed the presence of low levels of HSV-2 and HIV in these samples (Supplementary Figure 5), illustrating that at these sequencing depths, mNGS can be less sensitive than targeted techniques for very low-level infections. Interestingly, subject M051 had been receiving antiretroviral therapy until three weeks prior to presentation, and was found to have a CSF HIV viral load of 469 copies/mL by clinical testing. Manual review of mNGS reads for M051 showed that HIV reads were present, but below the thresholds used in this study (Supplementary Methods) [(15)](https://paperpile.com/c/aJnfHX/fjbtg).

*Search for novel viruses*

We tested analysis methods to improve the detection of divergent viruses that may not be well represented in standard reference libraries. We used the protein sequence classifier Kaiju on sequencing reads and BLASTx on assembled contigs to help identify more divergent sequences than could be detected by the DNA classifier KrakenUniq. We initially identified reads and contigs matching the putative Blacklegged-tick picorna-like virus 2 (BTPLV-2) in CSF from participant M015 (Supplementary Table 8 https://figshare.com/articles/dataset/Tables/13266506). The reference BTPLV-2 genome consisted of two contigs of length 1.1kb and 1.6kb that had been assembled from an environmental metagenomic survey of *Ixodes scapularis* ticks from Long Island, NY and Connecticut [(17)](https://paperpile.com/c/aJnfHX/9Usc7). We were able to *de novo* assemble two contigs of length 1.3kb and 4.5kb, which fully contained the published contigs. Interestingly, we only detected BTPLV-2 reads in one out of two independent extraction batches from this participant. We therefore performed a third extraction batch and found BTPLV-2 reads across multiple samples extracted in that batch, including the negative controls. Review of all samples sequenced in this study revealed a number of samples with low-level BTPLV-2 reads. Ultimately, we were not able to ascertain the source of the BTPLV-2 reads, but felt it unlikely to represent a true human pathogen.

**Case Vignettes**

**Four patients with Enterovirus Meningitis**

*Category: Infection, CSF PCR+; mNGS positive*

M007Z – 32-year-old pregnant woman at 40 weeks gestational age who presented with acute onset, severe headache, neck stiffness and daily fevers to a maximum of 38·6°C. On admission, she was treated empirically with vancomycin, meropenem due to a penicillin allergy, and acyclovir. CSF obtained on day of admission showed 1875 WBC (83% neutrophils), 5 RBC, total protein 107, and glucose 52. Labor was induced, and she delivered a healthy infant. She was diagnosed with enterovirus (EV) meningitis by CSF PCR, and antimicrobials were discontinued. Her neck stiffness and malaise improved, and fevers resolved by day 5. She reported episodic headaches at her 1-month and 6-month follow-up visits and was diagnosed with migraines.

M072E – 29-year-old woman with a history of migraines who developed an acute headache with photophobia, phonophobia and vomiting after a nonspecific gastrointestinal illness without diarrhea. She presented to the emergency department after 3 days of headache and reported no fevers or neck stiffness. CSF obtained on day of admission showed 91 WBC (92% lymphocytes), 2 RBC, total protein 43, and glucose 56. She was treated with ceftriaxone and acyclovir. She was diagnosed with EV meningitis by CSF PCR, and antimicrobials were discontinued. She reported resolution of all symptoms at discharge on day 2; no follow-up was available.

*Category: Unknown; mNGS positive*

M108A – 35-year-old pregnant woman at 7 weeks gestational age who had 4 weeks of fatigue and dull headache followed by acute worsening of headache, fever, and neck stiffness for 3 days prior to presentation. She was empirically treated with vancomycin, ceftriaxone and acyclovir. CSF obtained on day of admission showed 80 WBC (58% lymphocytes, 33% neutrophils), 3 RBC, total protein 53, and glucose 61. All antimicrobials were stopped after 48 hours and she was diagnosed with presumed viral encephalitis. She miscarried on day 3 of her hospitalization. The patient reported resolution of headache and neck symptoms by day 4 and reported being neurologically well at her 6-month appointment.

M126L – 29-year-old woman who experienced sudden onset nausea and vomiting, diffuse headache, neck stiffness and photophobia and sought medical care two days later with progressive headache. CSF obtained on the day of admission showed 116 WBC (54% neutrophils, 36% lymphocytes), 3 RBC, total protein 43, and glucose 56. Vancomycin and ceftriaxone were stopped at 24 hours, and acyclovir was stopped at 48 hours after CSF gram stain and cultures were negative. Herpes simplex virus type 1 (HSV-1) and herpes simplex virus type 2 (HSV-2) PCR returned as negative, respectively. She was diagnosed with presumed viral encephalitis. On day 3, she had improvement but not complete resolution of symptoms. She was readmitted to the emergency department 12 days later for continued headache, photophobia, and fatigue. CSF obtained in the emergency room showed 14 (95%) lymphocytes, total protein 33, and glucose 54. She did not receive further empiric antimicrobial treatment. She returned to the emergency department the following day due to pain from the lumbar puncture. She reported persistent fatigue at a 2-month follow-up visit.

**Conventional Laboratory Testing:** All subjects underwent CSF laboratory testing for HSV-1 and HSV-2 PCR, varicella-zoster virus (VZV) PCR, gram stain and culture, which were all negative. Only 1 patient did not have CSF VZV total/IgM serologies. Blood cultures and serum *B.burgdorferi* screens were sent in all cases and negative. Human immunodeficiency virus (HIV) antibody/antigen testing was checked during hospitalization or status was known within the six months prior to admission and negative. MRI brain or CT head was performed in three out of four cases, and no intraparenchymal findings were noted.

**CSF mNGS Results and Impact:** CSF mNGS testing was positive in two patients with PCR-confirmed enterovirus meningitis and another two patients who did not have a specific etiology identified by routine clinical testing and had not undergone clinical enterovirus PCR testing despite availability of the test. In all cases, patients were treated with broad-spectrum antibiotics until bacterial and herpes testing returned as negative; the outcome was favorable in all patients. Enteroviruses account for 55% of all viral meningitis cases and not surprisingly, patients are more likely to receive antimicrobial drugs when no cause is identified than those with a definitive diagnosis of EV meningitis [(18)](https://paperpile.com/c/aJnfHX/1JEVa). It is possible that testing for EV meningitis in M126L, as suggested in clinical guidelines [(19)](https://paperpile.com/c/aJnfHX/Cal44), may have prevented repeat emergency department admission and CSF testing.

**Progressive multifocal leukoencephalopathy (PML/AIDS co-infection) and detection of**  **JC Virus** **in immunocompromised hosts**

**PML:** *Category: Infection, CSF PCR+; mNGS positive*

M104W – 50-year-old man presented after a motor vehicle accident with right-sided weakness. After extensive testing, he was diagnosed with HIV-1 infection. On admission, his peripheral viral load was 21,3000 copies/ml and CD4+ T-cell count 34 cells/ul. CSF HSV, cytomegalovirus (CMV), Epstein-Barr virus (EBV), cryptococcal antigen and bacterial and mycobacterial cultures were negative. JC virus PCR in CSF was qualitatively positive. The patient clinically deteriorated and was made comfort measures only. Autopsy studies showed widespread demyelination and simian virus (SV) 40 inclusions in the cerebral cortex and cerebellum.

**Other:** *Category: Unknown; mNGS positive for JC virus in serum and/or urine*

M081N – 62-year-old man with psoriatic arthritis on methotrexate, adalimumab with a history of a splenectomy presented with fevers and myalgias of the proximal thigh muscles. Brain MRI showed periventricular and a single right middle cerebellar peduncle T2-hyperintense lesions without enhancement or diffusion restrictions. CSF showed 101 WBC (85% lymphocytes), and total protein 55 mg/dL. In a 2nd LP, CSF JC virus PCR was negative.

M106B – 70-year-old man with a remote history of colon cancer requiring a partial colectomy presented with worsening of gait instability, and progressive encephalopathy and fatigue. He was transferred to Massachusetts General Hospital after two hospitalizations at surrounding hospitals. He had multiple lumbar punctures with the first at an outside institution showing CSF WBC 454 cells, protein 243 mg/dL and glucose 118 mg/dL. The first CSF analyses at our hospital on hospital day 7 showed WBC 141 (19% lymphocytes), total protein 243 mg/dL, and glucose 118 mg/dL. Clinical testing for syphilis, *Listeria monocytogenes*, HSV, CMV, West Nile virus (WNV), *Borrelia burgdorferi* or *Borrelia miyamotoi* were negative. CSF JC virus PCR was not tested.

M127Q – 29-year-old man with common variable immune deficiency (CVID) maintained on monthly IVIG who presented after 10 days of a waxing and waning headache, neck pain and nausea. CSF showed 85 WBC (83% lymphocytes), protein 158 mg/dL, and glucose 28 mg/dL. CSF JC virus PCR was not tested.

**CSF mNGS Results and Impact:** CSF mNGS testing was positive for M104W possibly due to high viral burden in the setting of advanced HIV. The assembled genome from CSF using mNGS contained two repeats of the non-coding regulatory region (NCRR) associated with neurotropic variants [(20–22)](https://paperpile.com/c/aJnfHX/FflYZ+Bn0Xd+mGS3t). In contrast, in M081N and M106B, JC virus was detected in subjects’ urine but not in CSF by mNGS. The complete JC virus genome was assembled from urine from both subjects, and the configuration of its regulatory region resembled non-neurotropic variants. In 127Q, a non-neurotropic JC virus variant was detected in urine and serum, but not in CSF.

**Six patients with tick-borne infection**

**Powassan virus encephalitis**

*Category: Infection, other; mNGS positive*

M015B – The clinical case has been published for this 70-year-old woman with Powassan virus encephalitis, which was diagnosed by serology from CSF [(23)](https://paperpile.com/c/aJnfHX/ntM7w). mNGS was positive for Powassan virus from CSF that had been obtained approximately two weeks after symptom onset. The patient survived but had substantial neurological deficits.

M030T – The clinical case and mNGS results have been published for this 61-year-old man with Powassan virus encephalitis, which was detected by mNGS four weeks earlier than by conventional serology from CSF [(24)](https://paperpile.com/c/aJnfHX/IYTBA). The patient survived but had substantial residual neurological deficits.

*Category: Infection, other; mNGS negative*

M071C – 78-year-old man with history of subacute memory decline suspected due to vascular dementia who was admitted with gait instability, progressive somnolence, and fever to 40 degrees C. He had known tick exposures. MRI showed T2 flair hyperintensity in the bilateral thalami and susceptibility-weighted changes in the right thalamus, without enhancement. EEG showed generalized periodic discharges and right-lateralized periodic discharges, as well as generalized rhythmic delta slowing. CSF showed 18 WBC (77% PMN), total protein 58 mg/dL, and glucose 69 mg/dL. He was suspected to have viral meningoencephalitis and was treated with intravenous immunoglobulin (IVIG). He was diagnosed with Powassan virus by serology from CSF with positive enzyme-linked immunosorbent assay (ELISA) and plaque reduction neutralization test (PRNT) 1-4. mNGS was negative for Powassan virus from CSF that had been obtained 11 days after symptom onset. The patient was hospitalized for three weeks, including one week in the intensive care unit, and spent an additional month at a rehabilitation center. Nine months later, he had extrapyramidal symptoms and deficits in orientation and recall (Montreal Cognitive Assessment (MoCA) score 22/30) and was prescribed donepezil.

**Neuroborreliosis**

*Category: Infection, other; mNGS positive*

M089U – 63-year-old man with history of renal transplant (13 years previously, treated with prednisone 7·5mg daily) and post-transplant lymphoproliferative disease (11 years previously) who presented with several days of fever to a maximum of 38·2℃, headache, neck stiffness, and left arm paresthesias. He reported moderate outdoor activity for yard work. Lyme serology obtained three weeks previously had shown positive Western Blot with 2/3 IgM bands and 2/10 IgG bands; he had not received treatment. On the current admission, CSF studies showed 14 WBC (58% polymononuclear leukocytes), 46 RBCs, total protein 54 mg/dL, and glucose 63 mg/dL. Peripheral Lyme serology was again positive with 2/3 IgM bands and 4/10 IgG bands. Quantitative serology showed a Lyme-to-total-antibody ratio for IgM of >11·1 in serum and <1 in CSF; IgG was <1 in both (Imugen). mNGS was positive for *Borrelia burgdorferi* from a CSF sample obtained approximately three days after onset of neurological symptoms. He was treated with intravenous ceftriaxone for four weeks and had improved energy but mild persistent headache.

M101P – 51-year-old man with no prior medical history who developed bilateral eye pressure and severe visual impairment, and was found to have bilateral optic disc edema. MRI showed equivocal T2 hyperintense signal in the right optic nerve without definite enhancement. He was diagnosed with optic neuritis / papillitis. CSF studies obtained several weeks after symptom onset showed 91 WBCs (91% lymphocytes), 6 RBCs, total protein 65, glucose 55. Lyme serology was positive with IgM 3/3 bands and IgG 8/10 bands. CSF serology was consistent with intrathecal antibody production (Lyme-to-total antibody ratios for IgM were 1·6 in CSF and 3·3 in serum, and for IgG were 8·4 in CSF and 2·4 in serum). mNGS was positive for *Borrelia burgdorferi*. The patient was treated with intravenous ceftriaxone for four weeks and had an only modest improvement in symptoms, with preserved vision on the left but limited recovery on the right (20/200).

**Anaplasma phagocytophilum meningitis**

*Category: Infection, other; mNGS positive*

M017J – 48-year-old man with no significant past medical history who was admitted with two weeks of daily fevers to a maximum of 40 ℃. He had a headache, photophobia, and phonophobia. He reported significant time outdoors including gardening and camping throughout New England and had removed an engorged tick approximately two weeks prior to presentation. He was found to have thrombocytopenia (platelet nadir 47). He was treated with ceftriaxone and doxycycline and defervesced within 24 hours. CSF obtained on hospital day two showed 4 WBC (61% lymphocytes), total protein 42 mg/dL, and glucose 66 mg/dL. He was diagnosed with anaplasmosis by positive serum PCR. mNGS was positive for *Anaplasma phagocytophilum* from a CSF sample obtained approximately two weeks after symptom onset. The patient was treated with doxycycline and improved.

**Conventional Laboratory Testing:** All subjects underwent extensive testing that was negative for other infectious agents, including tick-borne co-infections. The three patients with Powassan virus infection had negative tests for Lyme disease, anaplasmosis, and babesiosis. The patient with anaplasmosis had a positive Lyme screening ELISA but negative Western Blot, a negative babesia smear, and negative Powassan virus serology from CSF. Of the patients diagnosed with Lyme disease, M089U had negative tests for anaplasma and babesia, while M101P had negative testing for other infectious causes of optic neuritis.

**CSF mNGS Results and Impact:** CSF mNGS testing was positive for two out of three patients with Powassan virus encephalitis in this study, and mNGS results did not seem related to the timing of sample collection relative to symptom onset. Powassan encephalitis is currently diagnosed by serology, with concern that viral nucleic acid may not be routinely detectable as for other arboviruses including WNV. However, our results suggest that nucleic-acid based testing may be useful for screening, particularly since a positive result could spare patients unnecessary further testing and empiric treatment. Similarly, we detected *Borrelia burgdorferi* reads in both of the patients in this study diagnosed with Lyme disease. CSF PCR is discouraged from use in diagnosing CNS Lyme disease due to low sensitivity, however, our results suggest that low levels of nucleic acid may be detectable using highly sensitive techniques. Our detection of *Anaplasma phagocytophilum* nucleic acid in CSF was unexpected since this is not commonly considered to be a cause of CNS infection. However, headache is a common symptom of anaplasmosis, and our findings raise the possibility that direct neurological involvement may be more common than currently appreciated.

**Eight patients with varicella zoster virus CNS disease**

*Category: Infection, CSF PCR positive; mNGS positive*

M134 – 39-year-old woman with depression but no other significant past medical history who presented with fever, headache, neck stiffness and cutaneous herpes zoster (DFA positive), and was referred to the ED by her PCP for meningitis evaluation. No imaging was obtained due to the lack of focal exam lesions. CSF on hospital day one showed 235 WBC (100% lymphocytes) prior to the initiation of IV acyclovir.

*Category: Infection, other; mNGS positive with use of methylated DNA depletion*

M049 – 81-year-old woman with schizoaffective disorder, atrial fibrillation, lung and bladder cancer, and recently diagnosed cutaneous herpes zoster who was admitted with altered mental status. MRI could not be performed due to patient confusion. Head CT showed no acute intracranial abnormalities. CSF on hospital day three showed 191 WBC (75% lymphocytes, 25% monocytes) following three days of PO famciclovir and three days of IV acyclovir.

M070 – 73-year-old woman with chronic obstructive pulmonary disease (COPD) with recent high dose steroids for exacerbation who presented to an outside hospital with dermatomal rash, progressive somnolence, and a seizure. Initial CSF showed 146 WBC and positive VZV by PCR, and treatment was initiated with acyclovir. Following a second seizure, the patient was transferred to our institution, and CSF on hospital day eight showed 33 WBC (94% lymphocytes, 5% monocytes, and 1% neutrophils) after eight total days of IV acyclovir.

*Category: Infection, other; mNGS negative*

M043 – 79-year-old man with chronic lymphocytic leukemia (CLL), COPD, and history of herpes zoster ophthalmicus who presented with new cranial nerve palsies and was admitted after identification of subacute stroke on follow up MRI. CSF on hospital day one showed 14 WBC (99% lymphocytes, 1% monocytes) in the setting of >6 weeks IV acyclovir and PO valacyclovir.

M069 – 77-year-old man with COPD, hypertension (HTN), hyperlipidemia (HLD), type 2 diabetes mellitus (T2DM), and coronary artery disease (CAD) after coronary artery bypass graft surgery (CABG) with a recent diagnosis of trigeminal neuralgia who presented with headache, dysphagia, ataxia, and dizziness and was admitted due to dehydration. CSF on hospital day five showed 105 WBC (93% lymphocytes).

M115 – 70-year-old man with HIV (VL ND, CD4 279), remote intravenous drug user (IVDU), hepatitis C (HCV) with cirrhosis, with resolved rash (with unspecified PO antiviral drug) three weeks prior and new onset of left arm weakness and neck pain, who was admitted after identification of expansile hemorrhagic lesion in cervical spine. CSF on hospital day three showed 300 WBC (80% lymphocytes).

M122 – 37-year-old man with recent history of Bell’s palsy empirically treated with valacyclovir who presented with subacute onset of vertigo, blurry vision, and hearing loss, and was admitted after MRI identified multiple enhancing cranial nerves. CSF on hospital day three showed 133 WBC (90% lymphocytes) after several weeks of valacyclovir and one day of IV acyclovir.

M123 – 85-year-old man with dementia, atrial fibrillation, hypertension, remote benign brain tumor resection, and recent cutaneous Zoster infection who presented with altered mental status and low grade fever, and was admitted for the question of possible seizures. CSF on hospital day one showed 14 WBC (98% lymphocytes) following several weeks of valacyclovir.

**Conventional Laboratory Testing:** All eight subjects underwent extensive laboratory testing including negative PCR or serology for HSV, Epstein-Barr virus (EBV), Lyme, Syphilis, and Mycobacterium tuberculosis (MTB). HIV testing was negative for all subjects with the exception of M115K, a subject with chronic HIV infection and found to have positive Mycoplasma IgG (1·7, normal <0·9) and IgM (1032; normal <770). Targeted VZV PCR from CSF was positive for M070 (performed at an outside hospital from an earlier CSF sample) and M134, and negative for the other six subjects. Total VZV CSF antibodies were detected for all subjects (>1:2) (M134 not tested); M115 also had positive IgM (>1:1). Two subjects (M069 and M123) developed skin lesions during their hospital admission that tested positive for VZV antigen.

**CSF mNGS Results and Impact:** CSF mNGS testing was positive for VZV by routine mNGS for M134, positive for M049 and M070 using enhanced methods, and negative for the other five subjects. The positive cases were all associated with acute onset of symptoms and had either no prior antiviral treatment or limited exposure, while the negative cases tended to be subacute to chronic infections, often with extensive antiviral treatments prior to the collection of CSF. Since alternative pathogens were not detected, treatment with IV acyclovir was continued for all subjects. With the exception of subject M123, who expired on hospital day 19 following a hospital course complicated by pulmonary edema and delirium, all other subjects were discharged and completed courses of IV acyclovir and/or PO valacyclovir with resolution of symptoms.

**Three patients with HIV-associated neurological syndromes**

*Category: Infection, other; mNGS positive*

M061 – 30-year-old man with no significant past medical history who presented with bifrontal headache, fever, and vomiting and was diagnosed with HIV-1 infection. On admission, his peripheral viral load was 518,000 and CD4+ T-cell count 167 cells/ul. CSF testing showed 81 WBC (95% lymphocytes), total protein 124 mg/dL, glucose 44 mg/dL and was obtained prior to antiretroviral therapy (ART) initiation.

*Category: Infection, CSF PCR positive; mNGS equivocal*

M051 – 65-year-old man with HIV-1 infection, off ART for an estimated three weeks, who presented with leg weakness and fatigue and was found to have renal failure. His plasma viral load was 163,000 copies/ml and CD4+ T-cell count 77 cells/ul. CSF testing showed 0 WBC, total protein 27 mg/dL, glucose 60 mg/dL and was obtained prior to ART reinitiation.

*Category: Infection, other; mNGS positive with use of hybrid capture*

M010 – 26-year-old woman with 2-3 months of abdominal pain, dysphagia and 20lb weight loss who presented with possible seizure versus syncopal episode and was diagnosed with HIV-1 infection. Her admission plasma viral load was 132,000 copies/ml and CD4+ T-cell count 22 cells/ul. Her brain MRI with contrast and EEG were normal. CSF testing showed 0 WBC, total protein 35mg/dL, glucose 46 mg/dL and was obtained prior to ART initiation.

**Conventional Laboratory Testing:** All three subjects underwent laboratory testing for opportunistic infections which included negative CSF gram stain and cultures, HSV, and venereal disease research laboratory (VDRL) for syphilis. Serological testing in blood included negative CMV IgG, Toxoplasmosis IgG/IgM, cryptococcal Ag and Trep-Sure enzyme immunoassay. M051 had a positive interferon-gamma release assay (IGRA) the year prior and was diagnosed with latent tuberculosis (TB) infection; the two other subjects had negative IGRA testing. Subject M051 had a CSF HIV-1 viral load of 469 copies/ml, and the two other subjects did not have CSF testing for HIV RNA.

**CSF mNGS Results and Impact:** CSF mNGS testing was positive for M061 in the setting of high plasma viral load and CSF pleocytosis, likely indicative of HIV meningitis. The other two patients did not have CSF pleocytosis. M051 was recently on ART, and had low levels of CSF HIV viremia, albeit higher than published limit of detection of 313 copies/ml [(15)](https://paperpile.com/c/aJnfHX/fjbtg) for HIV-1 in CSF for mNGS and clinical limit of detection of 20 copies/ml for HIV-1 by PCR. Metagenomic sequencing results were considered equivocal as only 1 read (26 kmers) was detected by standard mNGS, and even with some improvement by hybrid capture (10 reads with 74 kmers), this was below this study’s limit for reporting a positive result from mNGS. For patient M10, HIV was not detected in CSF by standard mNGS, but was detected with the addition of hybrid capture; the clinical relevance of this is uncertain since the patient did not have CSF pleocytosis or definitive neurological symptoms. No alternative pathogens were detected in any of the cases, and all patients improved with ART initiation.

**References**

1. [Gire SK, Goba A, Andersen KG, Sealfon RSG, Park DJ, Kanneh L, Jalloh S, Momoh M, Fullah M, Dudas G, Wohl S, Moses LM, Yozwiak NL, Winnicki S, Matranga CB, Malboeuf CM, Qu J, Gladden AD, Schaffner SF, Yang X, Jiang P-P, Nekoui M, Colubri A, Coomber MR, Fonnie M, Moigboi A, Gbakie M, Kamara FK, Tucker V, Konuwa E, Saffa S, Sellu J, Jalloh AA, Kovoma A, Koninga J, Mustapha I, Kargbo K, Foday M, Yillah M, Kanneh F, Robert W, Massally JLB, Chapman SB, Bochicchio J, Murphy C, Nusbaum C, Young S, Birren BW, Grant DS, Scheiffelin JS, Lander ES, Happi C, Gevao SM, Gnirke A, Rambaut A, Garry RF, Khan SH, Sabeti PC. 2014. Genomic surveillance elucidates Ebola virus origin and transmission during the 2014 outbreak. Science 345:1369–1372.](http://paperpile.com/b/aJnfHX/978Nz)

2. [Matranga CB, Andersen KG, Winnicki S, Busby M, Gladden AD, Tewhey R, Stremlau M, Berlin A, Gire SK, England E, Moses LM, Mikkelsen TS, Odia I, Ehiane PE, Folarin O, Goba A, Kahn SH, Grant DS, Honko A, Hensley L, Happi C, Garry RF, Malboeuf CM, Birren BW, Gnirke A, Levin JZ, Sabeti PC. 2014. Enhanced methods for unbiased deep sequencing of Lassa and Ebola RNA viruses from clinical and biological samples. Genome Biol 15:519.](http://paperpile.com/b/aJnfHX/gO7E8)

3. [Bolger AM, Lohse M, Usadel B. 2014. Trimmomatic: a flexible trimmer for Illumina sequence data. Bioinformatics 30:2114–2120.](http://paperpile.com/b/aJnfHX/FPa3s)

4. [Breitwieser FP, Baker DN, Salzberg SL. 2018. KrakenUniq: confident and fast metagenomics classification using unique k-mer counts. Genome Biol 19:198.](http://paperpile.com/b/aJnfHX/91u7i)

5. [Metsky HC, Siddle KJ, Gladden-Young A, Qu J, Yang DK, Brehio P, Goldfarb A, Piantadosi A, Wohl S, Carter A, Lin AE, Barnes KG, Tully DC, Corleis B, Hennigan S, Barbosa-Lima G, Vieira YR, Paul LM, Tan AL, Garcia KF, Parham LA, Odia I, Eromon P, Folarin OA, Goba A, Viral Hemorrhagic Fever Consortium, Simon-Lorière E, Hensley L, Balmaseda A, Harris E, Kwon DS, Allen TM, Runstadler JA, Smole S, Bozza FA, Souza TML, Isern S, Michael SF, Lorenzana I, Gehrke L, Bosch I, Ebel G, Grant DS, Happi CT, Park DJ, Gnirke A, Sabeti PC, Matranga CB. 2019. Capturing sequence diversity in metagenomes with comprehensive and scalable probe design. Nat Biotechnol 37:160–168.](http://paperpile.com/b/aJnfHX/r9Arq)

6. [Brister JR, Ako-Adjei D, Bao Y, Blinkova O. 2015. NCBI viral genomes resource. Nucleic Acids Res 43:D571–7.](http://paperpile.com/b/aJnfHX/rHHiJ)

7. [Bushnell B. 2016. BBMap.](http://paperpile.com/b/aJnfHX/CnYMm)

8. [Menzel P, Ng KL, Krogh A. 2016. Fast and sensitive taxonomic classification for metagenomics with Kaiju. Nat Commun 7:11257.](http://paperpile.com/b/aJnfHX/XR9to)

9. [Nurk S, Meleshko D, Korobeynikov A, Pevzner PA. 2017. metaSPAdes: a new versatile metagenomic assembler. Genome Research.](http://paperpile.com/b/aJnfHX/oDfMz)

10. [Altschul SF, Gish W, Miller W, Myers EW, Lipman DJ. 1990. Basic local alignment search tool. J Mol Biol 215:403–410.](http://paperpile.com/b/aJnfHX/CooFH)

11. [Mollerup S, Asplund M, Friis-Nielsen J, Kjartansdóttir KR, Fridholm H, Hansen TA, Herrera JAR, Barnes CJ, Jensen RH, Richter SR, Nielsen IB, Pietroni C, Alquezar-Planas DE, Rey-Iglesia A, Olsen PVS, Rajpert-De Meyts E, Groth-Pedersen L, von Buchwald C, Jensen DH, Gniadecki R, Høgdall E, Langhoff JL, Pete I, Vereczkey I, Baranyai Z, Dybkaer K, Johnsen HE, Steiniche T, Hokland P, Rosenberg J, Baandrup U, Sicheritz-Pontén T, Willerslev E, Brunak S, Lund O, Mourier T, Vinner L, Izarzugaza JMG, Nielsen LP, Hansen AJ. 2019. High-Throughput Sequencing-Based Investigation of Viruses in Human Cancers by Multienrichment Approach. J Infect Dis 220:1312–1324.](http://paperpile.com/b/aJnfHX/sHuQW)

12. [Asplund M, Kjartansdóttir KR, Mollerup S, Vinner L, Fridholm H, Herrera JAR, Friis-Nielsen J, Hansen TA, Jensen RH, Nielsen IB, Richter SR, Rey-Iglesia A, Matey-Hernandez ML, Alquezar-Planas DE, Olsen PVS, Sicheritz-Pontén T, Willerslev E, Lund O, Brunak S, Mourier T, Nielsen LP, Izarzugaza JMG, Hansen AJ. 2019. Contaminating viral sequences in high-throughput sequencing viromics: a linkage study of 700 sequencing libraries. Clinical Microbiology and Infection.](http://paperpile.com/b/aJnfHX/WFPop)

13. [Schlaberg R, Chiu CY, Miller S, Procop GW, Weinstock G, Professional Practice Committee and Committee on Laboratory Practices of the American Society for Microbiology, Microbiology Resource Committee of the College of American Pathologists. 2017. Validation of Metagenomic Next-Generation Sequencing Tests for Universal Pathogen Detection. Arch Pathol Lab Med 141:776–786.](http://paperpile.com/b/aJnfHX/OhScG)

14. [Blauwkamp TA, Thair S, Rosen MJ, Blair L, Lindner MS, Vilfan ID, Kawli T, Christians FC, Venkatasubrahmanyam S, Wall GD, Cheung A, Rogers ZN, Meshulam-Simon G, Huijse L, Balakrishnan S, Quinn JV, Hollemon D, Hong DK, Vaughn ML, Kertesz M, Bercovici S, Wilber JC, Yang S. 2019. Analytical and clinical validation of a microbial cell-free DNA sequencing test for infectious disease. Nature Microbiology.](http://paperpile.com/b/aJnfHX/Y8LJS)

15. [Miller S, Naccache SN, Samayoa E, Messacar K, Arevalo S, Federman S, Stryke D, Pham E, Fung B, Bolosky WJ, Ingebrigtsen D, Lorizio W, Paff SM, Leake JA, Pesano R, DeBiasi R, Dominguez S, Chiu CY. 2019. Laboratory validation of a clinical metagenomic sequencing assay for pathogen detection in cerebrospinal fluid. Genome Res 29:831–842.](http://paperpile.com/b/aJnfHX/fjbtg)

16. [Simner PJ, Miller HB, Breitwieser FP, Pinilla Monsalve G, Pardo CA, Salzberg SL, Sears CL, Thomas DL, Eberhart CG, Carroll KC. 2018. Development and Optimization of Metagenomic Next-Generation Sequencing Methods for Cerebrospinal Fluid Diagnostics. J Clin Microbiol 56.](http://paperpile.com/b/aJnfHX/Um4N7)

17. [Tokarz R, Sameroff S, Tagliafierro T, Jain K, Williams SH, Moses Cucura D, Rochlin I, Monzon J, Carpi G, Tufts D, Diuk-Wasser M, Brinkerhoff J, Ian Lipkin W. 2018. Identification of Novel Viruses in Amblyomma americanum , Dermacentor variabilis , and Ixodes scapularis Ticks. mSphere.](http://paperpile.com/b/aJnfHX/9Usc7)

18. [McGill F, Griffiths MJ, Bonnett LJ, Geretti AM, Michael BD, Beeching NJ, McKee D, Scarlett P, Hart IJ, Mutton KJ, Jung A, Adan G, Gummery A, Sulaiman WAW, Ennis K, Martin AP, Haycox A, Miller A, Solomon T, Adedeji A, Katharine A, David B, Thomas B, Antony C, David C, John C, Richard C, John C, Iain C, James D, Simon E, Camelia F, Peter F, Clive G, Katherine G, Shirley H, Kevin J, Matthew J, Ildiko K, Susan L, Karim M, Sarah M, Jane M, Kavya M, Martin M, Ed M, Christopher M, Monika P, Hassan P, Nikhil P, Haris R, Mark R, Amy R, Andrew R, Stefan S, Monty S, Philip S, Neil T, Alastair W, Martin W. 2018. Incidence, aetiology, and sequelae of viral meningitis in UK adults: a multicentre prospective observational cohort study. The Lancet Infectious Diseases.](http://paperpile.com/b/aJnfHX/1JEVa)

19. [Venkatesan A, Tunkel AR, Bloch KC, Lauring AS, Sejvar J, Bitnun A, Stahl J-P, Mailles A, Drebot M, Rupprecht CE, Yoder J, Cope JR, Wilson MR, Whitley RJ, Sullivan J, Granerod J, Jones C, Eastwood K, Ward KN, Durrheim DN, Solbrig MV, Guo-Dong L, Glaser CA, International Encephalitis Consortium. 2013. Case definitions, diagnostic algorithms, and priorities in encephalitis: consensus statement of the international encephalitis consortium. Clin Infect Dis 57:1114–1128.](http://paperpile.com/b/aJnfHX/Cal44)

20. [Pfister LA, Letvin NL, Koralnik IJ. 2001. JC virus regulatory region tandem repeats in plasma and central nervous system isolates correlate with poor clinical outcome in patients with progressive multifocal leukoencephalopathy. J Virol 75:5672–5676.](http://paperpile.com/b/aJnfHX/FflYZ)

21. [Ryschkewitsch CF, Jensen PN, Major EO. 2013. Multiplex qPCR assay for ultra sensitive detection of JCV DNA with simultaneous identification of genotypes that discriminates non-virulent from virulent variants. J Clin Virol 57:243–248.](http://paperpile.com/b/aJnfHX/Bn0Xd)

22. [Marzocchetti A, Wuthrich C, Tan CS, Tompkins T, Bernal-Cano F, Bhargava P, Ropper AH, Koralnik IJ. 2008. Rearrangement of the JC virus regulatory region sequence in the bone marrow of a patient with rheumatoid arthritis and progressive multifocal leukoencephalopathy. J Neurovirol 14:455–458.](http://paperpile.com/b/aJnfHX/mGS3t)

23. [Zachary KC, LaRocque RC, Gonzalez RG, Branda JA. 2019. Case 3-2019: A 70-Year-Old Woman with Fever, Headache, and Progressive Encephalopathy. N Engl J Med 380:380–387.](http://paperpile.com/b/aJnfHX/ntM7w)

24. [Piantadosi A, Kanjilal S, Ganesh V, Khanna A, Hyle EP, Rosand J, Bold T, Metsky HC, Lemieux J, Leone MJ, Freimark L, Matranga CB, Adams G, McGrath G, Zamirpour S, Telford S 3rd, Rosenberg E, Cho T, Frosch MP, Goldberg MB, Mukerji SS, Sabeti PC. 2018. Rapid Detection of Powassan Virus in a Patient With Encephalitis by Metagenomic Sequencing. Clin Infect Dis 66:789–792.](http://paperpile.com/b/aJnfHX/IYTBA)
